# Receptor Crosslinking: A General Method to Trigger Internalization and Lysosomal Targeting of Therapeutic Receptor:Ligand Complexes

**DOI:** 10.1038/mt.2015.178

**Published:** 2015-10-27

**Authors:** Paul R Moody, Edward J Sayers, Johannes P Magnusson, Cameron Alexander, Paola Borri, Peter Watson, Arwyn T Jones

**Affiliations:** 1 Cardiff School of Pharmacy and Pharmaceutical Sciences, Cardiff University, Cardiff, Wales; 2 School of Pharmacy, University of Nottingham, Nottingham, England; 3 School of Biosciences, Cardiff University, Cardiff, Wales

## Abstract

A major unmet clinical need is a universal method for subcellular targeting of bioactive molecules to lysosomes. Delivery to this organelle enables either degradation of oncogenic receptors that are overexpressed in cancers, or release of prodrugs from antibody–drug conjugates. Here, we describe a general method that uses receptor crosslinking to trigger endocytosis and subsequently redirect trafficking of receptor:cargo complexes from their expected route, to lysosomes. By incubation of plasma membrane receptors with biotinylated cargo and subsequent addition of streptavidin to crosslink receptor:cargo–biotin complexes, we achieved rapid and selective lysosomal targeting of transferrin, an anti-MHC class I antibody, and the clinically approved anti-Her2 antibody trastuzumab. These three protein ligands each target a receptor with a distinct cellular function and intracellular trafficking profile. Importantly, we confirmed that crosslinking of trastuzumab increased lysosomal degradation of its cognate oncogenic receptor Her2 in breast cancer cell lines SKBR3 and BT474. These data suggest that crosslinking could be exploited for a wide range of target receptors, for navigating therapeutics through the endolysosomal pathway, for significant therapeutic benefit.

## Introduction

For many therapeutics, delivery to lysosomes must be carefully controlled, either to minimize or to maximize proteolytic degradation of the therapeutic, and/or its target. For example, antibodies that bind to transferrin receptor (TfR) for delivery across the blood–brain barrier (BBB) must avoid lysosomal degradation.^1,2,3^ On the other hand, antibodies that target oncogenic receptors are often targeted toward lysosomes in order to provide therapeutic benefits, either by depleting the growth-inducing oncogenic receptors or by unleashing toxic drugs from antibody–drug conjugates (ADCs).^4^

In general, the first stage in directing ADCs to these environments conceptually involves taking the ADC to a cell and then exploiting the antibodies' specificity to bind a receptor that is selectively expressed on the diseased cell of choice.^4^ However, specific activity of the ADC within the target cell requires not just cell entry at a particular portal, but that the ADC:receptor complex traffics to lysosomes,^5^ where the cytotoxic drug can be released into the cytosol and access its target. This is either by degradation of the antibody or by cleavage of an antibody–drug linker.^6,7,8^ Inefficient lysosomal delivery, which in fact is evident for many ADCs,^9,10^ is expected to limit the amount of cytotoxic drug released inside tumor cells and result in suboptimal potency.^5^ To date, the only ADCs that have demonstrated sufficient efficacy to gain and retain clinical approval are trastuzumab–emtansine and brentuximab–vedotin.^11^

In order to evaluate delivery of exogenous proteins to lysosomes within the context of ADCs, we sought to exploit the enhanced trafficking to lysosomes that many receptors perform when clustered or crosslinked into “supramultivalent” interactions. This enhanced and sometimes aberrant lysosomal delivery has been observed for many receptors,^12^ including rabies G protein,^13^ ErbB family receptors such as epidermal growth factor receptor,^14,15^ acetylcholine receptors,^16,17^ and FcRn receptors.^18^ These findings were demonstrated in a range of cell types, including hamster kidney,^12^ mouse neuroblastoma,^13^ human kidney,^14^ human epidermal,^15^ rat muscle,^16^ *Xenopus* muscle,^17^ and human endothelial cells.^18^ Furthermore, crosslinking was induced in these reports by a range of methods, including streptavidin (SA),^12,17^ bivalent antibodies,^13,16,18^ natural ligands,^14,18^ and multivalent designed ankyrin repeat proteins (DARPins).^15^ In the case of CD20 receptors, antibody-mediated crosslinking has been utilized to modify cell signaling and drive apoptosis in myeloma cells.^19^

Surprisingly, despite the need for methods to deliver therapeutic ligands to lysosomes, the possibility of exploiting crosslinking for enhancing the uptake and subcellular targeting of therapeutic vectors and/or their cognate receptors has not been widely studied. Here, we demonstrate that we can increase delivery of three exogenously administered proteins, targeting distinct receptors, to lysosomes by formation of biotin: SA complexes at the plasma membrane. To do this, we add exogenous biotinylated antibodies or biotinylated protein ligands to cells and optionally induce complex formation with SA. By generating proteins that are dual-labeled with biotin and fluorophores, and imaging these by live cell confocal microscopy, we observe major differences in intracellular traffic of uncomplexed versus complexed proteins. As models to demonstrate this phenomenon, we selected three exogenous protein ligands that either do not traffic to lysosomes in their uncomplexed state (transferrin (Tf)) or do so minimally: the anti(MHC I) antibody W6/32 and the anti-Her2 antibody trastuzumab (TRz).

The trafficking route of Tf has been extensively characterized: It first binds to the TfR, and both then internalize together via clathrin-mediated endocytosis,^20^ which requires the AP2-coat complex.^21^ Following release of bound iron, Tf:TfR is recycled to the plasma membrane, where the Tf is then released.^22^ The ability of Tf to recycle has been exploited for delivery of various therapeutics (drugs, genes, proteins) across biological barriers including the BBB.^23,24^ TfR-mediated transport across the BBB occurs via transcytosis, in which TfR:cargo complexes are endocytosed at the apical face of endothelial cells and subsequently recycled at the distal basolateral surface. In addition to Tf, antibodies that bind TfR have been investigated for their ability to cross this barrier, but these efforts have been hindered by trafficking of TfR to lysosomes.^1,2,3^ An understanding of TfR:cargo trafficking may therefore enable us to design improved vectors for delivery of therapeutics into the brain via a transcytosis route that avoids lysosomal delivery. Other work on TfR trafficking has shown that local clustering of TfR increases the rate of endocytosis^25^ and that lysosomal delivery can be induced using a monoclonal bivalent anti-TfR antibody.^26^

The W6/32 antibody targets the MHC class I complex, which localizes predominantly to the plasma membrane.^27^ In contrast to Tf, endocytosis of the anti(MHC I) antibody W6/32 is clathrin independent.^28,29,30^ A “quality control” mechanism is proposed to exist,^31^ in which loss of β2 microglobulin from the MHC class I complex results in clustering of the remaining MHC class I heavy chain (which contains the epitope for W6/32)^32^ and its subsequent delivery to lysosomes.^33^ It has further been shown that induced crosslinking of the MHC class I complex causes signaling,^34,35,36,37^ which may serve as an indicator for the integrity of the MHC class I complex. Determining the endocytic fate of complexed W6/32 is thus a crucial test of the MHC class I quality control hypothesis.

TRz is a humanized monoclonal antibody that binds to Her2 and is used clinically either as an unconjugated antibody (“Herceptin”)^38^ or as the ADC TRz-emtansine (“Kadcyla”).^39^ Her2 is overexpressed in 15–20% of diagnosed breast cancers,^40,41^ where it forms oncogenic signaling dimers with other, ligand activated, receptors from the ErbB family. Her2 is considered to be difficult to deliver to lysosomes, firstly because it is resistant to internalization and secondly because the majority of endocytosed receptor recycles back to the plasma membrane.^38,42^ Furthermore, patients treated clinically with TRz routinely develop resistance to treatment, which prevents Her2 degradation.^43,44^ One promising method for overcoming resistance to internalization is to induce Her2 clustering,^45,46,47,48,49,50^ yet it remains to be determined whether SA can be used to deliver Her2:antibody complexes to lysosomes for degradation.

Here, we report that targeting cells with biotinylated ligands and subsequent addition of SA efficiently targets Tf, W6/32, and TRz to lysosomes and also enhances the degradation of Her2 in breast cancer cell lines that overexpress this receptor.

## Results

We initially investigated whether formation of biotin:SA complexes at the plasma membrane affects the endocytosis and traffic of the iron carrying protein Tf. Normally this protein undergoes endocytosis via the TfR into clathrin-coated vesicles, is trafficked to recycling endosomes, and then recycled back to the plasma membrane, thus avoiding delivery to lysosomes.^22,51^

**Figure 1:**
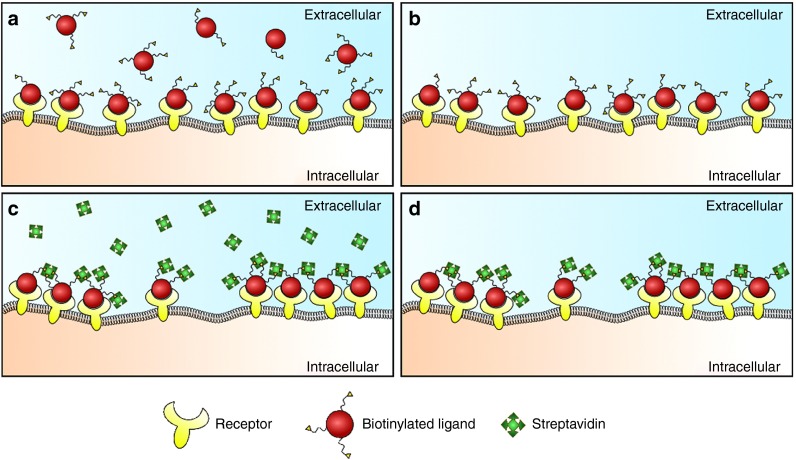
**Formation of protein–Bi:SA complexes by sequential incubation**. (**a**) Exogenous biotinylated protein is added to cells. (**b**) Excess unbound protein is removed by washing. (**c**) Streptavidin is added to cells, which has the capacity to cluster receptors by formation of extended crosslinks between receptor:ligand–biotin complexes. (**d**) Excess streptavidin is removed by washing prior to incubation at 37 °C. Cells are subsequently imaged by live cell confocal microscopy to monitor location of the complex in endolysosomal organelles.

Dual-labeled Tf (Tf-Bi-647) was generated by reaction of Tf-biotin (Tf-Bi) with NHS-Alexa647 (see **Supplementary Figure S2** for physical characterization and **Supplementary Tables S1** and **S2** for quantification). Due to the presence of multiple biotins per Tf-Bi-647 and multiple biotin-binding sites per SA, co-incubation of Tf-Bi-647 and SA to cells could result in formation of Tf-Bi-647:SA aggregates in solution.^52^ This was avoided by using a sequential addition protocol^53^ (**Figure 1**) that involved incubating HeLa cells with Tf-Bi-647, washing to remove unbound Tf-Bi-647, and then addition of SA. In order to prevent Tf-Bi-647 endocytosis before the addition of SA, incubations with Tf conjugates, SA, and SA conjugates were performed on ice.

**Figure 2:**
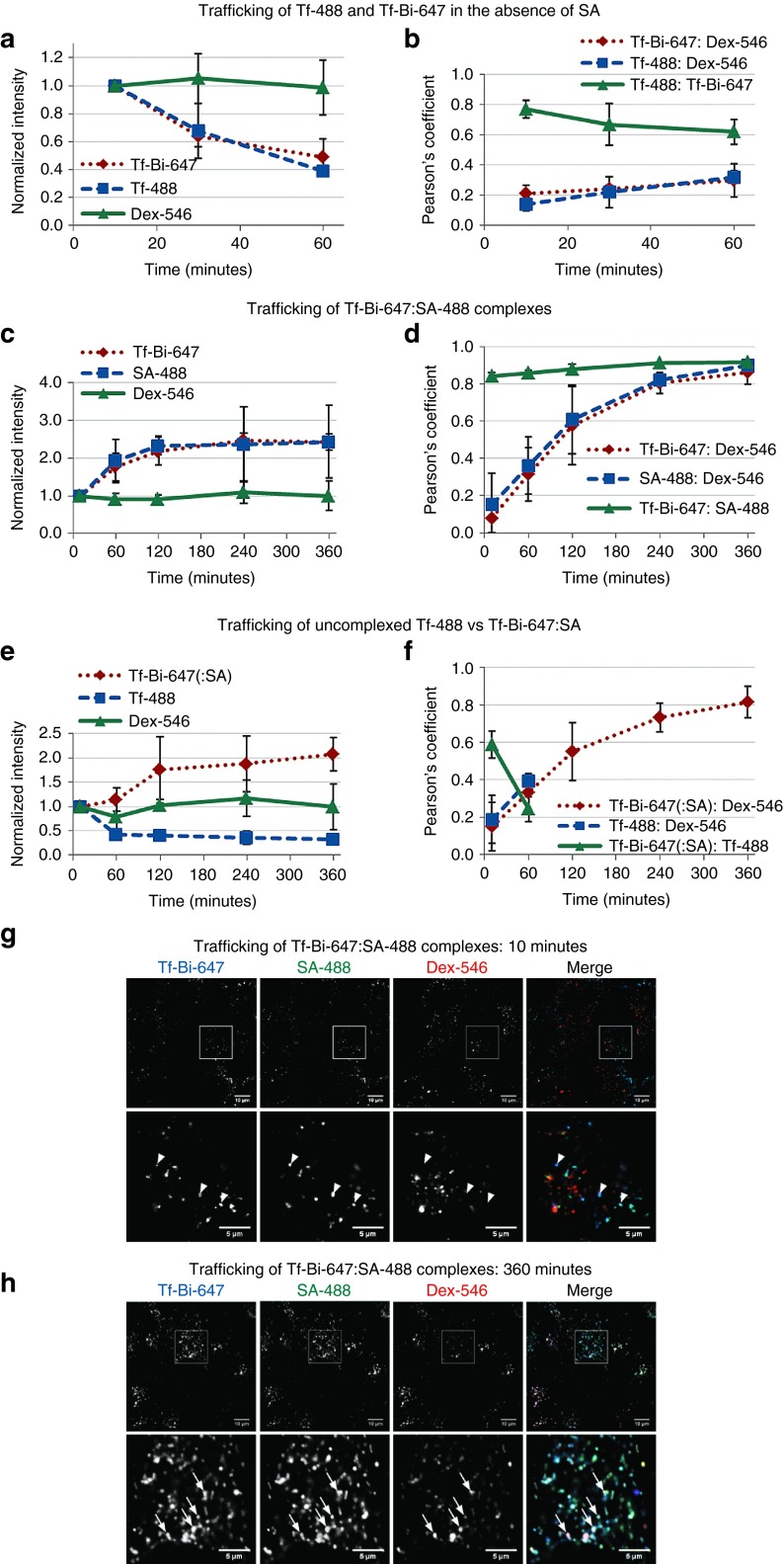
**Tf-Bi-647:SA complexes traffic to lysosomes**. HeLa cells were pulse-chased with Dex-546 to label lysosomes, and then incubated for 30 minutes in serum-free media. Cells were then placed on ice and incubated with the following solutions (15 min per incubation step, with PBS washes between each incubation): (**a** and **b**) co-incubation of 10 µg/ml Tf-Bi-647 and 10 µg/ml Tf-488, (**c,** **d,** **g,** and **h**) 10 µg/ml Tf-Bi-647, followed by incubation with 1 µg/ml SA-488. (**e** and **f**) co-incubation of 10 µg/ml Tf-Bi-647 and 10 µg/ml Tf-488, followed by incubation with 1 µg/ml unlabeled SA. After incubation on ice, cells were incubated at 37 °C in complete medium and imaged as live cells at the denoted time points. Normalized intensity (**a,** **c,** and **e**) represents the average fluorescence intensity per pixel above the threshold and after background subtraction (see **Supplementary Method 1**). Pearson's coefficients in **b**, **d**, and **f** are a measure of how closely the fluorescent regions of two images overlap. Error bars represent SD between mean values for three independent experiments. Representative single slice confocal microscopy images of Tf-Bi-647:SA complexes are shown after (**g**) 10 minutes or (**h**) 360 minutes. Arrowheads denote vesicles with Tf-Bi-647:SA-488 complexes, and arrows denote vesicles containing Tf-Bi-647, SA-488, and Dex-546. Scale bars of top rows (**g** and **h**) = 10 μm; bars of bottom rows (**g** and **h**) = 5 μm.

Initially, it was important to investigate whether biotinylation of Tf had any effect on its recycling, and for this, we simultaneously compared trafficking of biotinylated (Tf-Bi-647) and unbiotinylated (Tf-488) forms of this protein. HeLa cells were co-incubated with both forms on ice and then incubated at 37 °C for 60 minutes, with imaging of the two probes at the indicated time points by live cell confocal microscopy. Representative confocal microscopy images (**Supplementary Figure S3**) show that Tf-488 and Tf-Bi-647 are recycled. In order to quantify the rate of recycling, we calculated the average intensity per pixel in the fluorescent vesicle regions using a threshold and background subtraction method as described in **Supplementary Method 1** and **Figure S1**. We then defined the “normalized intensity” as the intensity relative to the mean fluorescence intensity at 10 minutes when the majority of Tf has been internalized into early and recycling endosomes. This dataset (*n* = 3) is quantified in **Figure 2a** which demonstrates that Tf-Bi-647 and Tf-488 are recycled out of cells at very similar rates, thus biotinylation does not affect the rate of Tf recycling.

In the same experiment, in order to evaluate lysosomal delivery of exogenously applied fluorescently labeled proteins, cells were pulse-chased with the fluid-phase endocytosis probe Dex-546 (see Materials and Methods) to specifically label lysosomes.^54^ Representative images demonstrate that Tf-488 and Tf-Bi-647 colocalize with each other, but not with Dex-546 labeled lysosomes (**Supplementary Figure S4**). To quantify this, colocalization between all pairs of fluorescent labels are represented using Pearson's coefficient (PC), which is calculated as the *r* value for the correlation of pixel intensities between corresponding pixels of two images. As expected, Tf-Bi-647 and Tf-488 colocalized together (mean PC of 0.65, **Figure 2b**, green line), and neither of these proteins colocalized strongly (PC < 0.35) with pulse-chased Dex-546 (**Figure 2b**). In summary, these results demonstrate that biotinylation of Tf did not perturb its endocytic traffic and Tf-Bi-647 is recycled rather than being delivered to lysosomes.

We then evaluated the capacity of SA-488 to bind Tf-Bi-647 on the plasma membrane. HeLa cells were labeled by sequential addition of Tf-Bi-647 and then fluorescently labeled SA (SA-488) at 0 °C. Labeled cells were then incubated at 37 °C for 6 hours with regular imaging of the two probes by live cell confocal microscopy. Quantitative analyses of these fluorescence images (**Figure 2c**,**d**) show an increase in normalized intensity for Tf-Bi-647 and SA-488 between 10 and 120 minutes (**Figure 2c**). This increase in the average intensity per pixel in the fluorescent regions above threshold suggests that the signal is clustered into fewer organelles. Strong colocalization of Tf-Bi-647 and SA-488 (PC > 0.8, **Figure 2d**, green line) suggests that they remained tightly associated for the duration of the experiment (6 hours). There was a clear time-dependent increase in colocalization of Dex-546 with both Tf-Bi-647 and SA-488 to a PC of ~0.9 (**Figure 2d**), demonstrating that Tf-Bi-647:SA-488 complexes did not effectively recycle and were trafficked to lysosomes. Representative images after 10 and 360 minutes of incubation for this experiment are shown in **Figure 2g**,**h**, which highlight the increased colocalization of Tf-Bi-647:SA complexes with Dex-546 in lysosomes between these time points.

We simultaneously compared lysosomal delivery of unbiotinylated Tf-488 and biotinylated Tf-Bi-647 in the presence of SA (**Figure 2e**,**f**). HeLa cells were first co-incubated at 0 °C with equal concentrations Tf-488 and Tf-Bi-647, washed, and then incubated with unlabeled SA. Unbiotinylated Tf-488 was rapidly recycled from the cell (**Figure 2e**, blue line), whereas Tf-Bi-647:SA complexes were retained within the cell (**Figure 2e**, red line) and trafficked to lysosomes (final PC > 0.8, **Figure 2f**). In **Figure 2f**, colocalization values are not shown for Tf-488 after 60 minutes, due to the fact that the majority of it has been recycled from the cell. Representative images of Tf-Bi-647(:SA) are shown in **Supplementary Figure S3** (right-hand column), which highlight that addition of SA inhibits recycling of Tf-Bi-647.

We then investigated whether the Tf-Bi-647:SA complexes formed at the plasma membrane are internalized, like Tf, via the canonical clathrin-mediated endocytosis route. To test this, an adapter protein subunit AP2µ2 (which is essential for clathrin-mediated endocytosis) was depleted in HeLa cells using siRNA.^55^ Three AP2µ2 targeting sequences were tested, and western blotting demonstrated that all three effectively depleted the protein (**Supplementary Figure S5a**). We then initially tested whether depleting AP2µ2 from cells selectively influenced Tf uptake over BSA-488, which is proposed to enter via a different route.^56^ Co-incubation of control and AP2μ2-siRNA–treated cells with Tf-647 and BSA-488 demonstrated that depletion of AP2μ2 caused retention of Tf-647 on the plasma membrane (**Supplementary Figure S5b**), but no visual effects were noted on the localization and uptake of BSA-488 between the two cell treatments.

**Figure 3:**
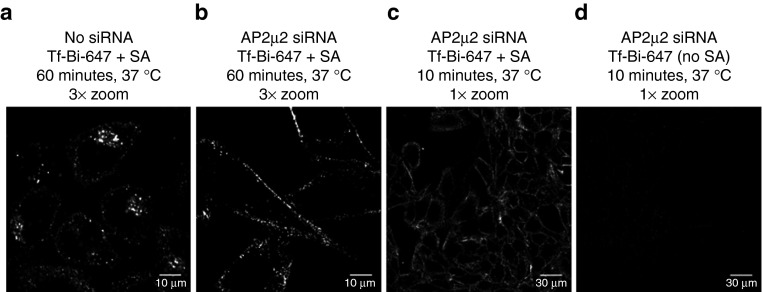
**Endocytosis of Tf-Bi-647:SA complexes is inhibited in AP2µ2-depleted cells**. HeLa cells were mock-treated with anti-GFP siRNA or treated with anti-AP2µ2 siRNA (see Materials and Methods). Cells were incubated at 0 °C with 20 µg/ml Tf-Bi-647 for 15 minutes, washed, then incubated at 0 °C with 1 µg/ml SA for 15 minutes. After washing, cells were incubated at 37 °C for the indicated time. All cells were acid washed (see Materials and Methods) immediately prior to imaging of live cells by confocal microscopy. Representative single slice images are shown. Bar = 10 μm (**a** and **b**) or 30 μm (**c** and **d**).

Tf-Bi-647:SA complexes were then generated on the surface of control and AP2µ2-depleted HeLa cells by sequential addition of Tf-Bi-647 and SA at 0 °C. Cells were then incubated at 37 °C for 10 or 60 minutes to permit internalization of the complexes. For removal of membrane-bound Tf-Bi-647:SA complexes, the cells were then acid washed at pH 4.5 prior to visualization by live cell confocal microscopy. Comparison of control cells (GFP-siRNA, **Figure 3a**) with AP2µ2-siRNA cells (**Figure 3b**) reveals that depletion of AP2µ2 results in retention of Tf-Bi-647:SA at the plasma membrane. This demonstrates that these complexes are, like Tf alone, endocytosed by clathrin-mediated endocytosis.

We additionally observed that after the acid wash procedure, Tf-Bi-647:SA complexes were still retained on the plasma membrane (**Figure 3c**), whereas Tf-Bi-647 alone was almost completely removed (**Figure 3d**). This suggests that Tf-Bi-647:SA complexes are refractory to acid washing and bound more tightly to cells due to increased avidity.

**Figure 4:**
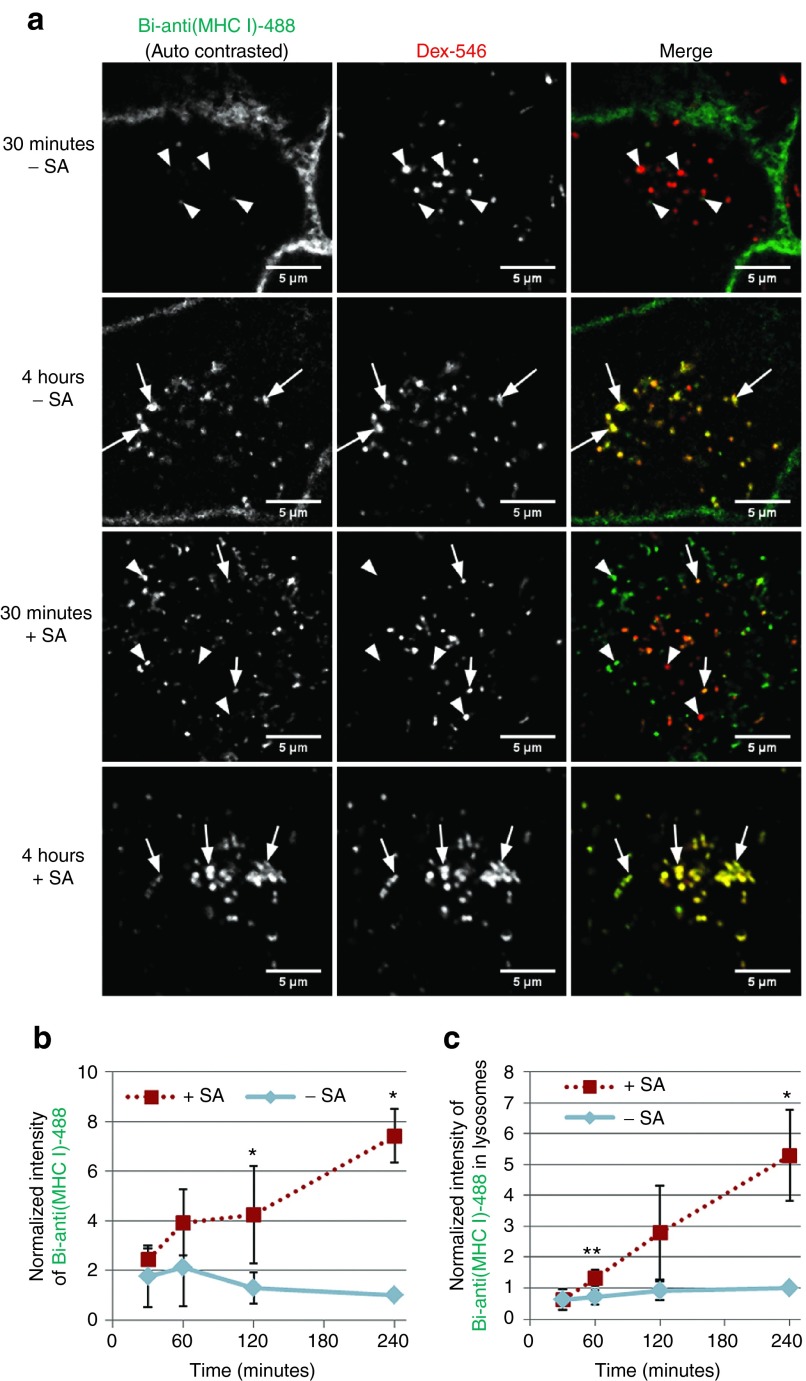
**SA induces delivery of Bi-anti(MHC I)-488 to lysosomes**. HeLa cells were pulse-chased with Dex-546 to label lysosomes, then labeled sequentially with Bi-anti(MHC I)-488, followed by 0 or 1 µg/ml SA. Cells were washed and then incubated at 37 °C for 240 minutes, with live cells imaged at the denoted time points by confocal microscopy. Five or more images were taken at each time point shown. (**a**) Representative single slice confocal images of Bi-anti(MHC I)-488 and Dex-546, ± SA, at 30-minute and 4-hour time points. Note that intensities in the left column have been enhanced postacquisition and so cannot be directly compared. Arrowheads denote non-colocalized vesicles and arrows denote colocalized vesicles. Bar = 5 μm. (**b**) Normalized intensity analysis of raw Bi-anti(MHC I)-488 images in the presence and absence of SA. All values are normalized relative to the intensity of Bi-anti(MHC I)-488 without SA at 240 minutes. (**c**) Bi-anti(MHC I)-488 intensity in lysosomes was calculated as total 488 fluorescence intensity in Dex-546-labeled regions, with corresponding background fluorescence intensity subtracted (see **Supplementary Method 2**). All values are normalized relative to the intensity of Bi-anti(MHC I)-488(:SA) at 240 minutes. Error bars represent SD between mean normalized values of three independent experiments. *P* values were calculated using one-tailed paired Student's *t*-test. *P < 0.05, **P < 0.01.

In view of the dramatic mislocalization of Tf caused by SA, we investigated whether a similar effect was observed for a very different ligand. For this, we selected the W6/32 antibody against the MHC class I receptor, which has been documented to enter cells via a clathrin-independent endocytic pathway.^28,29,30^ A biotinylated anti-MHC class I antibody was further labeled with NHS-Alexa488 to generate Bi-anti(MHC I)-488 (**Supplementary Figure S2** for physical characterization, **Supplementary Tables S1** and **S2** for quantification). HeLa cells with lysosomes preloaded with Dex-546 were incubated at 37 °C with Bi-anti(MHC I)-488 for 30 minutes, and then with 0 or 1 µg/ml unlabeled SA for 30 minutes. After treatment, cells were incubated at 37 °C for 4 hours, and then imaged by live cell confocal microscopy. For visual inspection, the contrast of each image was adjusted post-acquisition, to evaluate how the antibody is distributed throughout the cell (**Figure 4a**). In the absence of SA, there was extensive plasma membrane labeling, but a small fraction of the antibody had internalized into vesicles (indicated by arrowheads, top row). None of these internal structures were found to colocalize with lysosomal Dex-546. After 4 hours, plasma membrane labeling was still clearly evident, and some of the punctate structures were now located in lysosomes. Addition of SA gave a very different profile: Bi-anti(MHC I)-488 was localized entirely in punctate structures after 30 minutes, and after 4 hours, extensive colocalization was observed between the antibody and Dex-546. By examination of the wider fields of view for these images (**Supplementary Figure S6**), a complete lack of diffuse plasma membrane staining is evident for all cells treated with SA after only 30 minutes.

When the unprocessed images from these experiments were quantified, the results show that addition of SA results in significantly increased normalized intensity of Bi-anti(MHC I)-488 fluorescence within cells (**Figure 4b**; *P* < 0.001) at 240 minutes. From this, it was hypothesized that SA may increase the amount of Bi-anti(MHC I)-488 delivered to lysosomes. As PC colocalization values do not take total intensity into account, this hypothesis was tested by a different approach. The total amount of Bi-anti(MHC I)-488 in lysosomes was calculated by simultaneous imaging of Dex-546-labeled lysosomes and Bi-anti(MHC I)-488, then calculating the average fluorescence intensity of Bi-anti(MHC I)-488 in the regions that overlap with Dex-546 fluorescence (**Figure 4c**). SA caused a significantly increased delivery of Bi-anti(MHC I)-488 to lysosomes (*P* < 0.05) at 240 minutes. This increase in lysosomal delivery may be due to enhanced endocytosis of Bi-anti(MHC I)-488:SA complexes over Bi-anti(MHC I)-488 alone and/or an inhibition of its recycling.

The humanized monoclonal antibody TRz targets Her2, an oncogenic receptor tyrosine kinase that is overexpressed in a significant number of breast cancer patients. Based on our results on complexation of Tf and MHC class I with SA, we investigated whether complexation of biotinylated TRz with SA could enhance delivery of this antibody to lysosomes and if this could in turn degrade the Her2 receptor.

**Figure 5:**
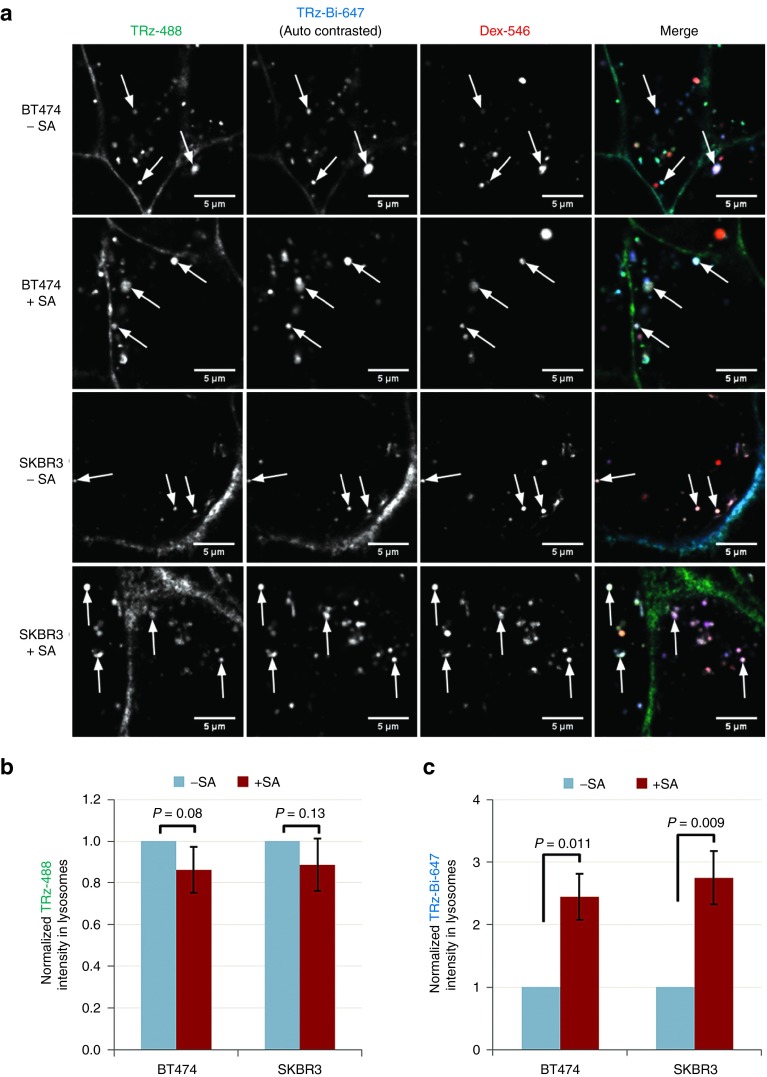
**SA selectively increases delivery of TRz-Bi-647 to lysosomes**. Dex-546 loaded SKBR3 and BT474 cells were co-incubated at 37 °C with TRz-488 and TRz-Bi-647, then with 0 or 1 µg/ml SA prior to incubation at 37 °C for 7 hours. Wash steps were included between each incubation. Single slice confocal microscopy images of the three fluorophores in live cells were then acquired. (**a**) Representative fluorescence microscopy images; arrows denote colocalization of Dex-546 with TRz-488. Contrast settings were automatically adjusted individually for each image. Bar = 5 μm. (**b**) TRz-488 or (**c**) TRz-Bi-647 intensity in lysosomes was calculated as total 488/647 fluorescence intensity in Dex-546-labeled regions, with corresponding background fluorescence intensity subtracted (see **Supplementary Method 2**). All values are normalized to the corresponding -SA condition. Bars represent SD between mean normalized values of three independent experiments. *P* values were calculated using one-tailed paired Student's *t*-test.

We generated TRz-Bi-647 by reaction of TRz with NHS-Alexa647 and NHS-biotin, and for an unbiotinylated control, we generated TRz-488 by reaction of TRz with NHS-Alexa488 (see **Supplementary Figure S2** for physical characterization and **Supplementary Table S1** and **S2** for quantification). The specificity of TRz-Bi-647 for Her2-expressing cells was confirmed in **Supplementary Figure S7**, which shows no association of TRz-Bi-647 with HeLa cells (which do not express Her2) and extensive binding of TRz-Bi-647 to the Her2-expressing cell lines SKBR3 and BT474 (ref. 38). As for the previous experiments, the lysosomes of these cells were labeled by pulse-chasing with Dex-546. Antibody trafficking was then evaluated by co-incubation with TRz-488 and TRz-Bi-647 for 30 minutes, followed by addition of 0 or 1 µg/ml SA prior to washing and incubating the cells for a further 7 hours at 37 °C. Live cell confocal microscopy analysis was performed at the end of this incubation period. For display of TRz-Bi-647 images only, contrast settings were adjusted for each image post-acquisition, so that the distribution of fluorescence can be seen both for low-intensity and for high-intensity images. For both fluorescent TRz variants, delivery to lysosomes was observed through colocalization with Dex-546. Comparative analysis showed that in both cells lines, in the presence or absence of SA, the localization and intensity of TRz-488 was unchanged, and the antibody was located both on the plasma membrane and in intracellular vesicles (**Figure 5a** left-hand column, wider views in **Supplementary Figure S8** left-hand column). However, in both cell lines, addition of SA caused TRz-Bi-647 to redistribute from the plasma membrane to vesicular structures (**Figure 5a** second column, wider views in **Supplementary Figure S8** second column). The data therefore indicate that SA:biotin complexation selectively increased the total amount of TRz-Bi-647 that was delivered to lysosomes within this 7-hour period. This hypothesis was tested quantitatively as described for Bi-anti(MHC I)-488, by using Dex-546 channel images to determine lysosomal regions, and then determining the total TRz intensity in these regions (**Figure 5b**,**c**). This demonstrated that SA significantly increased delivery of biotinylated TRz-Bi-647 to lysosomes by 145% in BT474 cells and 175% in SKBR3 cells. In contrast, lysosomal delivery of unbiotinylated TRz-488 was not significantly altered by addition of SA. As a further internal control, changes in the intensity of TRz-488 and TRz-Bi-647 in lysosomes were calculated from the same set of three-channel (488/546/647 nm) images.

**Figure 6:**
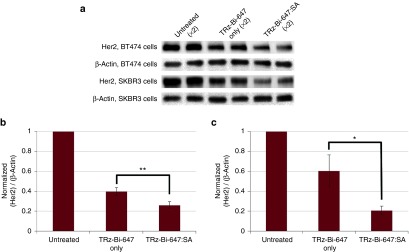
**Delivery of TRz-Bi-647 to lysosomes downregulates total Her2 levels**. SKBR3 and BT474 cells were incubated sequentially for 30 minutes at 37 °C with 0 (untreated) or 50 nmol/l TRz-Bi-647, then for 30 minutes at 37 °C with 0 or 1 µg/ml SA, followed by incubation at 37 °C for 7 hours. Wash steps were included between each incubation. Cell lysates were harvested and probed for total Her2 and β-actin levels by western blotting and chemiluminescence detection. Incubations and analysis were repeated in duplicate on three separate cell passages. (**a**) Representative immunoblotting from a single experiment performed in duplicate. (**b** and **c**) Analysis of Her2 intensities relative to β-actin in (**b**) BT474 cells (**c**) SKBR3 cells relative to a β-actin control. Bars represent SD, and *P* values represent variation between the mean of three independent experiments. *P < 0.05, **P < 0.01.

As TRz-Bi-647:SA enhanced lysosomal delivery in BT474 and SKBR3 cells, we investigated whether this caused increased degradation of Her2 and a concomitant reduction in total Her2 levels. For this, we lysed both these cell types at the end of the TRz-Bi-647 +/- SA incubation period and detected Her2 by western blotting (**Figure 6a**). As has previously been reported for unconjugated TRz,^57,58,59^ TRz-Bi-647 alone caused a decrease in Her2 levels that was decreased further in cells treated with both TRz-Bi-647 and SA. Quantitative analysis of this data (**Figure 6b**,**c**) reveals that treatment with TRz-Bi-647:SA complexes caused a significant decrease in Her2 levels (to 34% in BT474 cells, 66% in SKBR3 cells) relative to treatments with TRz-Bi-647 alone. In order to evaluate the duration of Her2 depletion, we used TRz-488 as a marker and confocal microscopy, to measure the amount of Her2 at the plasma membrane of SKBR3 and BT474 cells at various time intervals after the addition of SA to induce crosslinking and depletion. The images and quantification of the data are shown in **Supplementary Figure S9**. These demonstrate that plasma membrane Her2 levels had recovered to those of untreated cells 48 hours after crosslinking-induced depletion, consistent with a previous *in vivo* study.^45^

## Discussion

In this study, we have demonstrated for three distinct protein ligands that formation of receptor:[protein ligand]:SA complexes drives internalization and endocytic trafficking to lysosomes. We propose that this is a common response to receptor crosslinking and a mechanism with wide-ranging implications especially within the field of biomolecular targeting and delivery of biotherapeutics.

We provide evidence that SA crosslinks multiple biotinylated ligands on the plasma membrane and that this has a significant influence on the way they are processed by the cell within the endolysosomal system. The fact that membrane-bound Tf-Bi-647:SA complexes are resistant to acid washing suggests that a network of Tf ligands may be formed through SA crosslinking at the plasma membrane. A multivalent network is likely to have increased avidity for TfRs, and this crosslinking model is consistent with multiple reports of membrane receptors that traffic to lysosomes when crosslinked.^12,13,14,15,16,17,18,60,61,62^ Here, we propose that crosslinking of exogenous proteins on the cell membrane could be used as a general strategy for enhancing delivery of therapeutics to lysosomes.

Although evidence for crosslinking is provided, the extent of complex formation is unclear. The dependence of Tf-Bi-647:SA uptake on AP2µ2 suggests that crosslinked Tf still requires the clathrin machinery to mediate its endocytosis. Clathrin-coated vesicles are normally 60–120 nm in diameter,^63^ which suggests that crosslinked Tf does not form clusters larger than this. Further electron microscopy or single-molecule total-internal reflection fluorescence data will be required to determine the size and distribution of the complexes formed at the plasma membrane and to further characterize early and late endocytic events.

The data provided in this study add further evidence to a hypothesis that endocytosis and lysosomal delivery are common responses to clustering of receptors and that these are independent of the internalization mechanism. The MHC class I receptor has been shown to enter cells via a clathrin-independent route involving the small GTPase Arf6 (refs. 28,29,30). These studies showed that once internalized, the same W6/32 antibody is delivered to late endosomes or recycled. Here, we show that biotinylation of this antibody and addition of SA enhanced delivery of the antibody:receptor complex to lysosomes. This supports the previously stated hypothesis that clustered MHC class I molecules are targeted for degradation as part of a quality control mechanism that monitors the integrity of [MHC class I]:[β2 microglobulin] complexes.^31^

The disruption of recycling presented here may have important implications for transcytosis of therapeutic cargo across biological barriers. Within this field, a great deal of investment has focused on exploiting Tf and the TfR for delivery across the BBB.^23,24^ Our observations raise the possibility that current attempts to deliver drugs across the BBB using multivalent systems are compromised by trafficking to lysosomes, thus significantly reducing the fraction that is transcytosed to the brain parenchyma. It has been reported that transcytosis of anti-TfR antibodies from the apical to the basolateral component of the BBB may be enhanced by using antibodies with low receptor affinities^1^ or reduced valency to the TfR.^2,3^ In both cases, enhanced transcytosis may be caused by reduced receptor crosslinking.

Crosslinking could be a valuable strategy for improving delivery of ADCs to lysosomes. In order to optimize the proof-of-principle strategy described in this paper for therapeutic purposes, a range of improvements can be considered. For example, immunogenicity could be minimized by using less immunogenic variants of SA.^64^ Alternatively, crosslinking could be achieved by administration of multiple antibodies that bind to different epitopes of the same receptor.^46,47,48^ This is the likely mechanism underpinning the MARIANNE trial,^65^ in which the anti-Her2 ADC Kadcyla is dosed simultaneously with Pertuzumab, a second anti-Her2 antibody that binds a nonoverlapping epitope to that of Kadcyla.^66^ Based on our observations, it is expected that simultaneous addition of these two antibodies will induce synergistic crosslinking of Her2 receptors, resulting in lysosomal delivery of Kadcyla and release of its cytotoxic payload. It is likely that many potential ADCs underperform due to the fact that the receptor:ADC complex does not internalize efficiently. Furthermore, complexes that do internalize may recycle back to the plasma membrane rather than traffic to lysosomes, which is the target organelle for optimal drug release.

Although TRz drives downregulation of Her2 *in vitro*,^57,58,59^ TRz alone is insufficient to reduce Her2 levels *in vivo*, because tumors develop resistance to this treatment.^43^ We demonstrate for the first time that a Bi:SA crosslinking strategy causes a greater reduction in Her2 levels than treatment with TRz-Bi-647 alone. Further work will be required to determine if this is sufficient to overcome resistance to Her2 degradation *in vivo*. In support of this hypothesis, a previous report showed that a similar TRz-Bi:SA system induced endocytosis of the antibody *in vitro* and *in vivo*.^45^ Our results further strengthen this work by demonstrating both lysosomal delivery of TRz-Bi:SA complexes and degradation of the receptor.

An established method for drug delivery is to package drugs into nanoparticles that are coated with ligands, in order to enable selective binding to a target receptor and thus the target cell.^67^ Based on the data in this paper, we predict that the presence of multiple receptor-binding ligands on the surface of a nanoparticle is likely to induce receptor crosslinking, which may result in enhanced endocytosis and lysosomal delivery of nanoparticle:receptor complexes. Careful consideration should therefore be made when designing the number of receptor binding ligands on a nanoparticulate system.

The strategy described herein could be applied to a range of oncogenic receptors. Our data indicate that crosslinking of receptors using SA has broad potential for cancer therapy, by enabling improved subcellular targeting of ADCs and by driving degradation of oncogenic receptors. It remains to be determined how ubiquitous this response is among plasma membrane proteins, but the data provide strong pointers toward how designed ligand:receptor clusters could be utilized to alter cell trafficking pathways for therapeutic gain. In turn, by specifically avoiding receptor clustering, it may be possible to direct biomolecules away from degradative pathways, leading to pathway switching by design for new pharmaceutical entities.

## Materials and Methods

***Materials.*** Transferrin-Alexa488 (Tf-488), transferrin-Alexa647 (Tf-647), 10 kDa dextran-Alexa546 (Dex-546), SA-Alexa488 (SA-488), BSA-Alexa647 (BSA-647), Dulbecco's Modified Eagle's Medium (DMEM), phenol red-free DMEM with 25 mmol/l 4-(2-hydroxyethyl)piperazine-1-ethanesulfonic acid (HEPES), trypsin/ethylenediaminetetraacetic acid, and serum were purchased from Fisher Scientific (Loughborough, UK). Transferrin-biotin (Tf-Bi), unlabeled recombinant SA, bovine serum albumin (BSA), and all other chemicals were purchased from Sigma Aldrich (Gillingham, UK) unless otherwise stated.

***Generation of Tf-Bi-647.*** Lyophilized Tf-Bi (5 mg) suspended in 1 ml phosphate-buffered saline (PBS) pH 7.4, was added directly to 1 mg NHS-Alexa647 (Fisher Scientific) and reacted for 1 hour at room temperature to generate Tf-Bi-647. The conjugate was purified from unreacted Alexa647 into PBS pH 7.4 using a G-50 sephadex gel filtration column (Life Technologies, Paisley, UK).

***Generation of Bi-anti(MHC I)-488.*** Biotinylated anti-MHC class I antibody W6/32 (50 μg in 100 μl; eBioscience, Hatfield, UK) was reacted with 0.1 mg tetrafluorophenyl-Alexa488 (two vials from a TFP-Alexa488 labeling kit; Life Technologies) for 1 hour at room temperature, then purified by gel filtration into PBS pH 7.4, as per the kit instructions.

***Generation of TRz-Bi-647.*** TRz formulation (containing 21 mg/ml TRz, l-histidine HCl, l-histidine, α,α-trehalose dehydrate, and polysorbate 20) was kindly donated by the Velindre Cancer Centre (Cardiff, UK). This patient TRz formulation (1.5 ml, 32 mg TRz) was buffer exchanged into PBS pH 7.4, by sequential use of two 10 ml Zeba Spin desalting columns (Fisher Scientific). From the 1.7 ml of TRz that was eluted, 1.6 ml (30 mg) was added directly to 1 mg NHS-Alexa647, and the other 100 µl was retained for generation of TRz-488 (see below). NHS-biotin, 1 mg (Fisher Scientific) was solubilized in 1 ml of PBS pH 7.4, and 400 µl of this was immediately added to the solution of TRz and NHS-Alexa647 (final volume 2 ml). The mixture was left to react for 1 hour at room temperature without agitation to generate TRz-Bi-647. The conjugate was purified using two 10 ml Zeba Spin desalting columns (1 ml sample per column) in PBS pH 7.4, then filtered using a 0.2 µm filter (Millipore, Nottingham, UK).

***Generation of TRz-488.*** TRz, 100 µl in PBS pH 7.4, was reacted with 0.1 mg tetrafluorophenyl-Alexa488 (two vials from a TFP-Alexa488 labeling kit; Life Technologies) for 1 hour at room temperature and then purified by gel filtration into PBS pH 7.4, as per the kit instructions.

***Characterization of conjugated proteins.*** After purification, conjugated proteins were aliquotted into PCR tubes, frozen in liquid nitrogen, and stored at −20 °C prior to analysis. UV-visible absorbance spectra were obtained using a Jasco V-650 UV-Vis spectrophotometer. Biotin concentrations were calculated using a Sensolyte HABA Biotin Quantification Kit (Anaspec, Seraing, Belgium).

***Cell culture and sources.*** All cell lines were obtained from ATCC and routinely tested for mycoplasma infection. Cell lines were maintained as a subconfluent monolayer in complete medium: DMEM supplemented with 10% (v/v) heat-inactivated fetal calf serum (Gibco, Fisher Scientific). Cells were maintained in a humidified 5% CO_2_ incubator at 37 °C. For live cell imaging, cells were seeded onto 35 mm imaging dishes (MatTek, Ashland, MA), and for western blotting, cells were seeded into a six-well plate. For siRNA transfection studies, 150,000 cells were seeded per dish per well, and for all other studies, cells were seeded to 80–90% confluency. HeLa cells were left for a minimum of 16 hours to adhere, and SKBR3 and BT474 cells were left for a minimum of 36 hours to adhere. With the exception of incubations performed on ice, all incubations of live cells were performed in a humidified 37 °C, 5% CO_2_ incubator.

***Dex-546 labeling of lysosomes.*** Lysosomes were labeled by pulse-chasing with 200 μg/ml of the fluid-phase endocytic probe Dex-546 (ref. 54). For this, cells were pulsed with Dex-546 in complete medium, and the probe was subsequently chased in dextran-free complete medium. HeLa cells were pulsed for 4 hours and chased for 16 hours. SKBR3 and BT474 cells were pulsed for 14 hours and chased over the duration of the experiment.

***Sequential labeling of HeLa cells with Tf-Bi-647 and SA.*** HeLa cells were incubated for 30 minutes in serum-free medium (phenol red-free DMEM pH 7.4, containing 25 mmol/l HEPES, supplemented with 1 mg/ml BSA) to allow recycling of serum-derived transferrin. Cells were placed on ice for 15 minutes to inhibit endocytosis, incubated with 20 µg/ml Tf-Bi-647 in ice-cold serum-free medium for 15 minutes and then washed 3× in ice-cold PBS pH 7.4. Cells were then incubated with 0 or 1 µg/ml SA in ice-cold serum-free medium, and then washed 3× in PBS pH 7.4. Cells were finally incubated in pre-warmed imaging medium (phenol red-free DMEM pH 7.4, containing 25 mmol/l HEPES supplemented with 10% (v/v) heat-inactivated fetal calf serum) and analyzed at the indicated time-points by live cell confocal microscopy.

***Sequential labeling of HeLa cells with Bi-anti(MHC I)-488 or BT474/SKBR3 cells with TRz-Bi-647.*** Cells were treated with a total of 50 nmol/l antibody in imaging medium for 30 minutes at 37 °C, then washed 3× in PBS pH 7.4. This was followed by incubation with 0 or 1 µg/ml SA in imaging medium for 1 hour at 37 °C, and then washed 3× with PBS pH 7.4. After subsequent incubation in imaging medium for the indicated time periods, cells were either imaged by live cell confocal microscopy or were lysed for western blotting.

***Microscopy.*** Cells were analyzed on a Leica SP5 confocal laser-scanning microscope equipped with a 488 nm Ar laser, 543/633 nm HeNe laser, 63 × 1.4 NA objective utilizing Leica Type F immersion oil. A 1.5 times zoom was used except where otherwise indicated, producing a pixel size of 160 × 160 nm through a 95.5 μm pinhole. Alexa488, Alexa546, and Alexa647 were excited using 488, 543, and 633 nm lasers, respectively. Line-by-line generated images were acquired by simultaneous excitation at 488 and 633 nm, followed by excitation at 543 nm (15 ms per line). Single slice images of cells were taken ~1 µm above the coverslip. For each displayed time point, ≥5 distinct fields of view (each containing ~5 cells) were imaged. All microscopy imaging was performed on live cells.

***Microscopy analysis: colocalization.*** Within a field of view, cell–cell variation in the total amount of fluorescence intensity could cause unrepresentatively low PC values. To avoid this, regions of interest from individual cells were manually selected using the DIC image as a guide, while ensuring that the selected area was a minimum of 2,000 square pixels. PC values were calculated for each cell using the JaCOP ImageJ plugin.^68^ PC values were obtained for ≥8 cells per time point, and the mean values plotted. SD, where shown, was calculated for the variation between mean values of three independent experiments.

***Microscopy analysis: calculation of normalized intensities.*** A “normalized intensity” value was calculated to quantify how the average fluorescence intensity per pixel of fluorescent vesicle regions changes over time. Full details are provided in **Supplementary Method 1**. Briefly, an automated script was written to detect fluorescent regions and calculate the average fluorescence intensity per pixel in these regions. SD, where shown, was calculated for the variation between mean values of three independent experiments.

***Microscopy analysis: calculation of normalized intensity in lysosomes.*** The amount of protein delivered to lysosomes was estimated by determining the amount of labeled protein fluorescence in lysosomal regions, and full methodological details are provided in **Supplementary Method 2**. Briefly, fluorescence microscopy images of Dex-546 and labeled protein were captured simultaneously; the Dex-546 image was used to identify lysosomal regions, and mean protein fluorescence intensity within these lysosomal regions was then calculated. SD, where shown, was calculated for the variation between mean values of three independent experiments.

***siRNA depletion of AP2μ2 in HeLa cells.*** Cells were transfected with 100 nmol/l siRNA targeting AP2μ2 (5′-AAGUGGAUGCCUUUCGGGUCA-3′) or GFP (5′-GGCUACGUCCAGGAGCGCA-3′) using oligofectamine (Life Technologies) as described previously.^69^ Experimental conditions for achieving and evaluating siRNA depletion are provided in **Supplementary Methods 3 and 4**. siRNA-treated cells were labeled with Tf-Bi-647:SA complexes and incubated in imaging medium as described above. Cells were washed 3× in ice-cold PBS pH 7.4, before incubating with ice-cold acid wash solution (500 mmol/l NaCl, 50 mmol/l MES (4-morpholineethanesulfonic acid), pH 4.5) for 2 minutes to remove surface-bound protein.^67^ Cells were washed 3× in PBS pH 7.4, placed in imaging medium and then immediately analyzed by confocal microscopy.

***Western blotting.*** A detailed description for blotting and analysis is given in **Supplementary Method 5**. After blotting onto polyvinylidene fluoride membranes, receptors were probed using the following primary antibodies: Her2 (2242; Cell Signaling, Danvers, MA), AP2μ2 (611351; BD Bioscience, Oxford, UK), clathrin heavy chain (610499; BD Bioscience), glyceraldehyde 3-phosphate dehydrogenase (2118S; Cell Signaling), or β-actin (AC-15; Sigma Aldrich). Primary antibodies were detected with a corresponding horseradish peroxidase conjugated secondary antibody (Fisher Scientific). Horseradish peroxidase was probed using Clarity^TM^ Western Enhanced Chemiluminescence substrate (Bio-Rad, Hemel Hempsted, UK) and detected using a ChemiDoc XRS system (Bio-Rad).

***Statistical analysis.*** For all statistical analysis, data were obtained from three independent experiments (*n* = 3), and significance values were calculated using a paired Student's *t*-test.

**SUPPLEMENTARY MATERIAL** **Figure S1.** Automated calculation of normalized intensity. **Figure S2.** UV-visible absorbance spectrum of synthesized protein conjugates. **Figure S3.** Recycling of Tf-488 and Tf-Bi-647 and cellular retention of Tf-Bi-647(:SA) in HeLa cells. **Figure S4.** After 10 minutes of internalization, Tf-488 and Tf-Bi-647 colocalize together, but not with lysosomes. **Figure S5.** Depletion of AP2μ2 by siRNA and inhibition of Tf uptake in HeLa cells. **Figure S6.** Internalization of Bi-anti(MHC I)-488 complexes in HeLa cells is dramatically enhanced by addition of SA. **Figure S7.** TRz-Bi-647 selectively binds to cells that express Her2. **Figure S8.** SA selectively increases delivery of TRz-Bi-647 to lysosomes. **Figure S9.** Rate of recovery of Her2 at the plasma membrane, following depletion with TRz-Bi-647 and SA. **Table S1.** Analysis of UV-visible spectra. **Table S2.** Analysis of biotinylation quantification. **Supplementary Method 1.** Calculation of normalized intensities. **Supplementary Method 2.** Calculation of normalized Intensity in lysosomes. **Supplementary Method 3.** siRNA depletion of AP2μ2 in HeLa cells. **Supplementary Method 4.** Evaluation of siRNA depletion of AP2μ2. **Supplementary Method 5.** Western blotting and immunodetection. **Supplementary Method 6.** Recovery of Her2 at the plasma membrane following depletion with TRz-Bi-647 and SA.
