# Supplementary material for: Receptor Crosslinking: A General Method to Trigger Internalization and Lysosomal Targeting of Therapeutic Receptor:Ligand Complexes
Source: Mol Ther. 2015 Oct 27;23(12):1888–98. doi: 10.1038/mt.2015.178 (PMC4700114; doi:10.1038/mt.2015.178)
Supplement: Supplementary Information [file mt2015178x1.doc]

**Supplementary Material**

Title:

Receptor crosslinking – A General Method to Trigger Internalisation and Lysosomal Targeting of Therapeutic Receptor:Ligand Complexes

Authors:

Paul R. Moody, Edward J. Sayers, Johannes Magnusson, Cameron Alexander, Paola Borri, Peter Watson, Arwyn T. Jones

**Table of contents**

Supplementary Material – Methods

Supplementary Method 1. Calculation of Normalised Intensities S2

Supplementary Method 2. Calculation of Normalised Intensity in Lysosomes S2

Supplementary Method 3. siRNA depletion of AP2μ2 in HeLa cells S2

Supplementary Method 4. Evaluation of siRNA depletion of AP2μ2 S3

Supplementary Method 5. Western blotting and immunodetection S3

Supplementary Method 6. Recovery of Her2 at the plasma membrane following

depletion with TRz-Bi-647 and SA S4

Supplementary Material - Physical characterisation of conjugates

Physical characterisation of purified conjugate stock solutions S6

Calculation of degree of biotinylation S8

Supplementary Material – Figures S9

Supplementary Material – References S16

**Supplementary Material - Methods**

Supplementary Method 1. Calculation of Normalised Intensities

Confocal microscopy images of live cells were acquired, and each field of view (≥5 for each timepoint) was analysed using an ImageJ script without manual intervention. Pixels with intensity above an ImageJ Li^1^ threshold were selected as “fluorescent”, and the mean intensity of these fluorescent pixels was calculated. “Background” pixels were selected as those that were both below a Li threshold and spatially separated by at least 5 pixels from any pixel above this threshold. The mean background intensity was then calculated. Examples of fluorescent and background pixels selected by this method are shown in Supplementary Fig. 1. The mean background intensity value was subtracted from the mean fluorescence intensity value to give the corrected intensity. Corrected intensity values were calculated for ≥5 images at each timepoint, and the mean of these values was calculated. Mean corrected intensity values were normalised against an appropriate time point for that experiment, to give the Normalised Intensity value. Normalised Intensity values were calculated for 3 independent experiments, and error bars represent the standard deviation between these 3 experiments.

Supplementary Method 2. Calculation of Normalised Intensity in Lysosomes

For each field of view (≥5 for each timepoint), images of Dex‑546 and the dual-labelled protein ligand were acquired simultaneously by confocal fluorescence microscopy. Each field of view was then analysed using an ImageJ script without manual intervention. Background fluorescence intensity for the labelled protein was calculated as described for Normalised Intensities. Lysosomal regions were identified by applying a Li threshold to the Dex‑546 channel image. This was used to select lysosomal regions from the labelled protein image, and the mean labelled fluorescence in these lysosomal areas was then calculated. The mean background intensity value was subtracted from the mean fluorescence intensity in lysosomes, to give the corrected fluorescence intensity in lysosomes. Corrected intensity values were calculated for ≥5 images, and the mean of these values was calculated. Mean corrected intensity values were normalised relative to an appropriate control value, to give the Normalised Intensity in Lysosomes. The mean Normalised Intensity in Lysosomes from 3 independent experiments is plotted; error bars represent the standard deviation across these 3 values. p values were calculated using a student’s t‑test (1-tailed, unequal variance).

Supplementary Method 3. siRNA depletion of AP2μ2 in HeLa cells

After plating cells onto 35 mm wells or 35 mm MatTek dishes, cells were incubated for 16-20 hr prior to transfection with 100nM siRNA targeting AP2μ2 (either AP2-a, 5’‑AAGUGGAUGCCUUUCGGGUCA-3’,^4^ AP2-b,

5’‑AGUGGAUGCCUUUCGGGUCA-3’, AP2-c, 5’-GUGGAUGCCUUUCGGGUCA-3’, or GFP siRNA 5’-GGCUACGUCCAGGAGCGCA-3’)^2^ using oligofectamine (Life Technologies) as described previously^2^. Briefly, per well of a six well plate, 100 pmols siRNA was diluted in OptiMEM (Life Technologies) to a final volume of 185 μL, while 2 μL oligofectamine was diluted in OptiMEM to a final volume 15 μL. Both solutions were gently mixed together and allowed to complex at room temperature for 30 min. Cells were washed and the growth medium replaced with 800 μL OptiMEM. To this, 200 μL of the siRNA complex was added dropwise onto the cells, which were then incubated for 4 hr. Subsequently, 500 μL of OptiMEM containing 30% FCS was added to each dish/well and the cells returned to the incubator for a further 48 hr before microscopy or Western blotting.

Supplementary Method 4. Evaluation of siRNA depletion of AP2μ2

After siRNA treatment, depletion of AP2μ2 was confirmed for each siRNA by Western blotting (Supplementary Fig. 6a). Disruption of AP2-dependant endocytosis following treatment with AP2-a and GFP siRNA was evaluated by microscopy (Supplementary Fig. 6b). For microscopy, siRNA-treated HeLa cells were incubated for 30 min in serum-free medium followed by co incubation with 100 μg/mL BSA-Alexa488 and 10 μg/mL Tf‑647 for 15 min at 37 °C. Cells were washed, the medium was replaced with imaging medium, and then the samples were immediately imaged by confocal microscopy.

Supplementary Method 5. Western blotting and immunodetection

Cells were seeded onto a 6-well plate, and treated as described for the given experiment. After treatment, cells were placed on ice, washed 3x in ice cold PBS and incubated with ice-cold lysis buffer (50 mM Tris-HCl, 150 mM NaCl, pH 8.0, 1% Triton X-100 containing cOmplete protease inhibitor cocktail (Roche)). Cells were incubated on ice on a shaking platform for 10 min and harvested into Eppendorf’s. The lysates were centrifuged at 13,000 × g (4°C) for 10 min and supernatants corresponding to 15 - 25 µg protein were then mixed with 4x SDS sample buffer containing 1mM DTT, heated at 95°C for 5 min and separated by SDS-PAGE.

Following electrophoresis, proteins were transferred to PVDF membranes and blocked with 5% Marvel Milk in PBS with 0.025% Tween-20. The membranes were probed with antibodies recognising Her2 (2242, Cell Signalling), AP2μ2 (611351, BD Bioscience), clathrin heavy chain (610499, BD Bioscience), GAPDH (2118S, cell signalling), or β-actin (AC-15, Sigma Aldrich), in PBS with 0.025% Tween-20. Primary antibodies were then probed with a corresponding HRP‑conjugated secondary antibody (Fisher Scientific) in PBS with 0.025% Tween‑20. HRP activity was detected using Clarity^TM^ Western Enhanced Chemiluminescence substrate (BioRad) in PBS with 0.025% Tween-20 and detected using a ChemiDoc XRS system (Biorad).

Her2 Western blots were quantified using ImageJ software. The mean intensity for each band was measured using a fixed area, and the mean surrounding background intensity was subtracted, to give the corrected intensity. The corrected intensity of each Her2 band was then divided by the corrected intensity of the corresponding β‑actin loading control. These [Her2]:[loading control] ratios were averaged for each condition, and then normalised to the ratio for control untreated cells. A student’s t-test (1-tailed, paired) was performed on these normalised sample:control ratios obtained from 3 independent experiments.

Supplementary Method 6. Recovery of Her2 at the plasma membrane following depletion with TRz-Bi-647 and SA

SKBR3 or BT474 cells were plated onto 35mm MatTek Dishes and incubated for 48 hr. Treated cells were subsequently incubated at 37°C with 1 µg/mL TRz‑Bi‑647, then with 1 µg/mL SA (in imaging medium), prior to incubation at 37°C for 6, 24 or 48 hrs. One dish was prepared of each cell type that was not treated with TRz‑Bi‑647 or SA. All dishes were then incubated with 1 µg/mL TRz‑488 for 1 hr in imaging medium prior to live cell confocal microscopy. Samples were washed 3x in PBS after each antibody or SA incubation. Single slice confocal microscopy images of TRz-488 and TRz-Bi-647 in live cells were then acquired as described in the main methods (Microscopy) and analysed using ImageJ (Microscopy analysis - Calculation of Normalised Intensities).

**
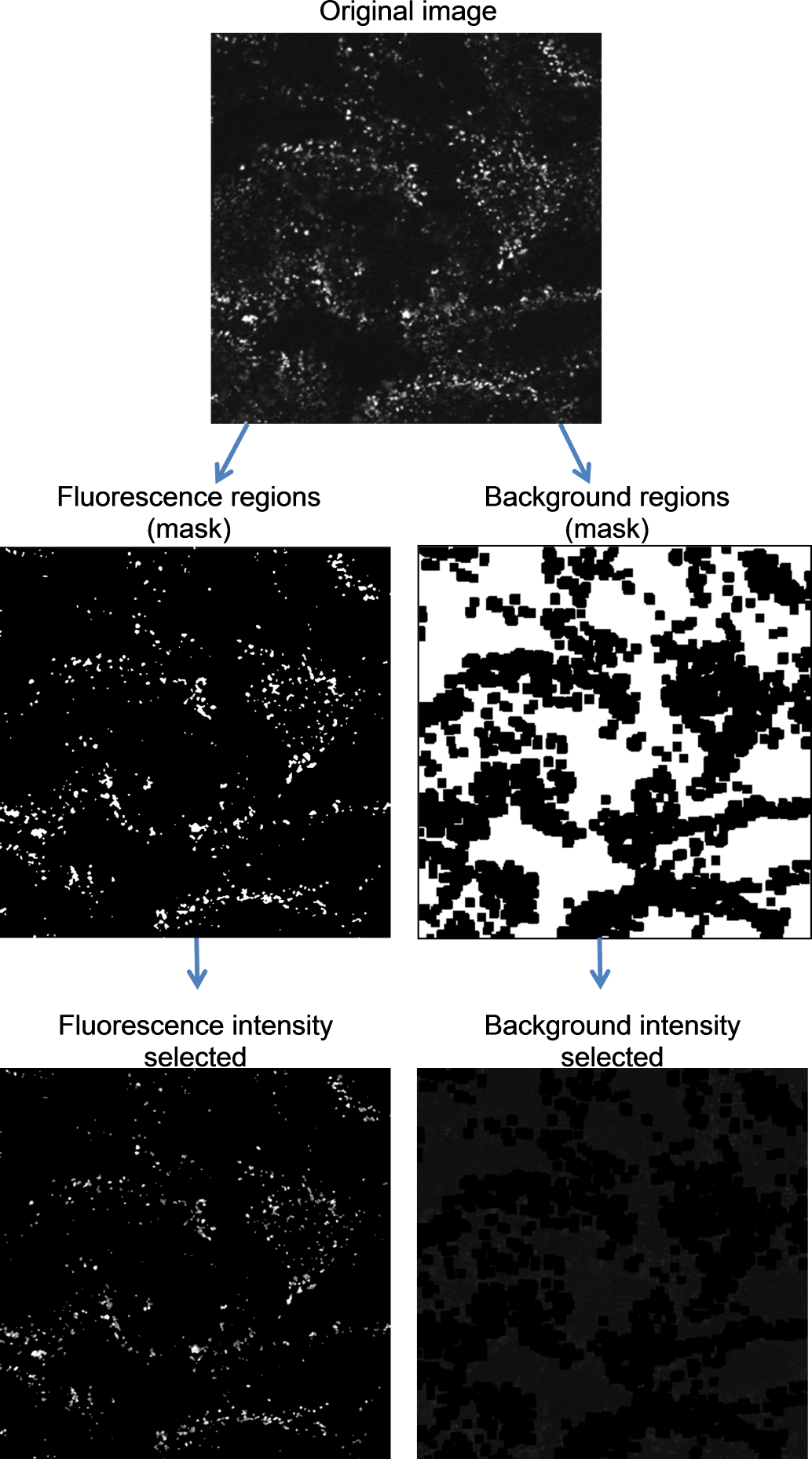
**

**Supplementary Figure S1. Automated calculation of Normalised Intensity**

An example of “fluorescence” and “background” regions selected for analysis of normalised intensity. The regions from which intensity is selected are shown in white.

**Supplementary Material - Physical characterisation of conjugates**

Physical characterisation of purified conjugate stock solutions

For each fluorescent protein conjugate synthesized and purified by gel permeation chromatography, the concentrations of protein and attached fluorophore were calculated *via* UV-visible spectroscopy. Spectra are shown in Supplementary Fig. 2 and analysed in Table 1. After measuring the spectrum of a diluted sample, absorbance peak heights were identified, and the hypothetical peak height of the undiluted stock was calculated. The contribution of fluorophore absorbance at 280 nm (manufacturers information) was then subtracted (A_494_ x 0.11 for Alexa 488 and A_650_ x 0.03 for Alexa 647). Protein concentrations were calculated from the 280 nm absorbance, using the reported extinction coefficients for diferric transferrin^3^ or for antibodies (200,000 Mˉ¹ cmˉ¹). Fluorophore concentrations were calculated from measured absorbances (the peak at 494 nm for Alexa-488 or at 650 nm for Alexa‑647) and the extinction coefficients provided by the manufacturer.

**a - Tf-Bi-647 (1 in 50 dilution) b - Bi-anti(MHC I)-488 (1 in 50 dilution)**


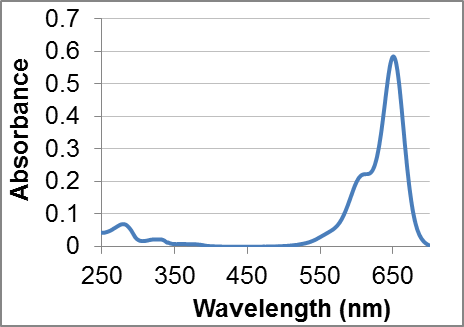

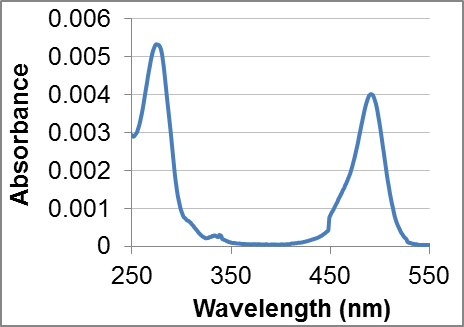


**c - TRz-Bi-647 (1 in 50 dilution) d - TRz-488 (1 in 50 dilution)**


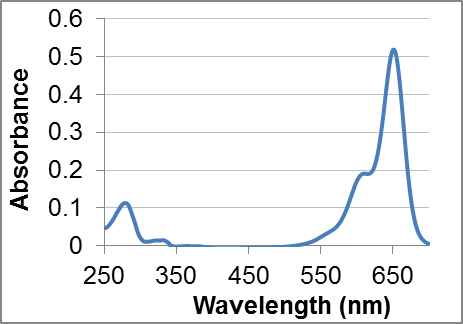

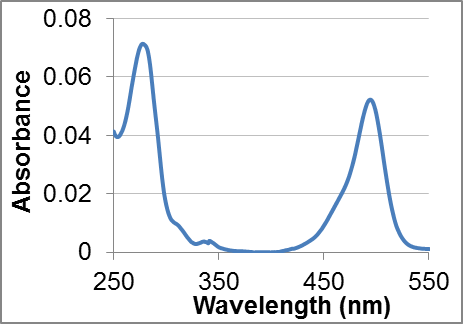


**Supplementary Figure S2 – UV-visible absorbance spectrum of synthesized protein conjugates**

Stock solutions of (a) Tf‑Bi‑647, (b) Bi-anti(MHC I)-488, (c) TRz-Bi-647, (d) TRz-488 were diluted 1 in 50 into PBS and analysed using a Jasco V-650 UV-visible spectrophotometer.

**Supplementary** Table 1 – Analysis of UV-visible spectra

|  | **Tf-Bi-647** | | **Bi-anti(MHC I)-488** | |
| --- | --- | --- | --- | --- |
|  | **Protein (280 nm)** | **Alexa 647 (650 nm)** | **Protein (280 nm)** | **Alexa 488 (494 nm)** |
| Dilution factor | 1 in 50 | | 1 in 50 | |
| Extinction coefficient (Mˉ¹) | 104,000 | 239,000 | 200,000 | 71,000 |
| Peak height of diluted sample | 0.068 | 0.58 | 0.0053 | 0.0041 |
| Peak height, corrected for dilution (x 50) | 3.40 | 29.0 | 0.265 | 0.205 |
| Peak heights, corrected for fluorophore absorbance at 280 nm | 2.53 | 29.0 | 0.242 | 0.205 |
| Concentration of stock (µM) | 24.0 | 121 | 1.21 | 2.89 |
| **Mean number of dyes per protein** | **5** | | **2.4** | |

**Supplementary Table 1 (continued) – Analysis of UV-visible spectra**

|  | **TRz-Bi-647** | | **TRz-488** | |
| --- | --- | --- | --- | --- |
|  | **Protein (280 nm)** | **Alexa 647 (650 nm)** | **Protein (280 nm)** | **Alexa 488 (494 nm)** |
| Dilution factor | 1 in 50 | | 1 in 50 | |
| Extinction coefficient (Mˉ¹) | 200,000 | 239,000 | 200,000 | 71,000 |
| Peak height of diluted sample | 0.11 | 0.52 | 0.071 | 0.052 |
| Peak height, corrected for dilution (x 50) | 5.5 | 26 | 3.55 | 2.6 |
| Peak heights, corrected for fluorophore absorbance at 280 nm | 4.72 | 26 | 3.26 | 2.6 |
| Concentration of stock (µM) | 23.6 | 109 | 16.3 | 36.6 |
| **Mean number of dyes per protein** | **4.6** | | **2.2** | |

Calculation of degree of biotinylation

Biotin concentrations were measured using a HABA Biotin Quantification Kit (Anaspec), in which HABA absorbance is enhanced by addition of biotin or biotin conjugates. A calibration curve for HABA absorbance was generated using known concentrations of free (unconjugated) biotin, which was used to convert HABA absorbance into biotin concentrations for our conjugate stock solutions (Supplementary Table 2). By combining the protein concentration calculated from 280 nm absorbance with the concentration of biotin on these proteins, the mean number of biotins per protein was calculated for stock solutions of Tf‑Bi‑647 and TRz-Bi-647. For Bi‑anti(MHC I)‑488, the manufacturer reports 3‑7 biotin molecules per antibody.

**Supplementary Table 2 – Analysis of biotinylation quantification**Sample absorbance values are the mean of 3 readings.

|  | **Tf-Bi-647** | **TRz-Bi-647** |
| --- | --- | --- |
| Dilution of stock before analysis | 1 in 2 | 1 in 2 |
| Mean absorbance of diluted sample | 0.980 | 0.717 |
| Biotin concentration of diluted sample, from a calibration curve (µM) | 64.65 | 45.29 |
| Biotin concentration of stock (µM) | 129.3 | 90.6 |
| Protein concentration (µM) (from UV-vis spectroscopy) | 24.0 | 23.6 |
| **Mean number of biotins per protein** | **5.4** | **3.8** |

**Supplementary Material - Figures**

**
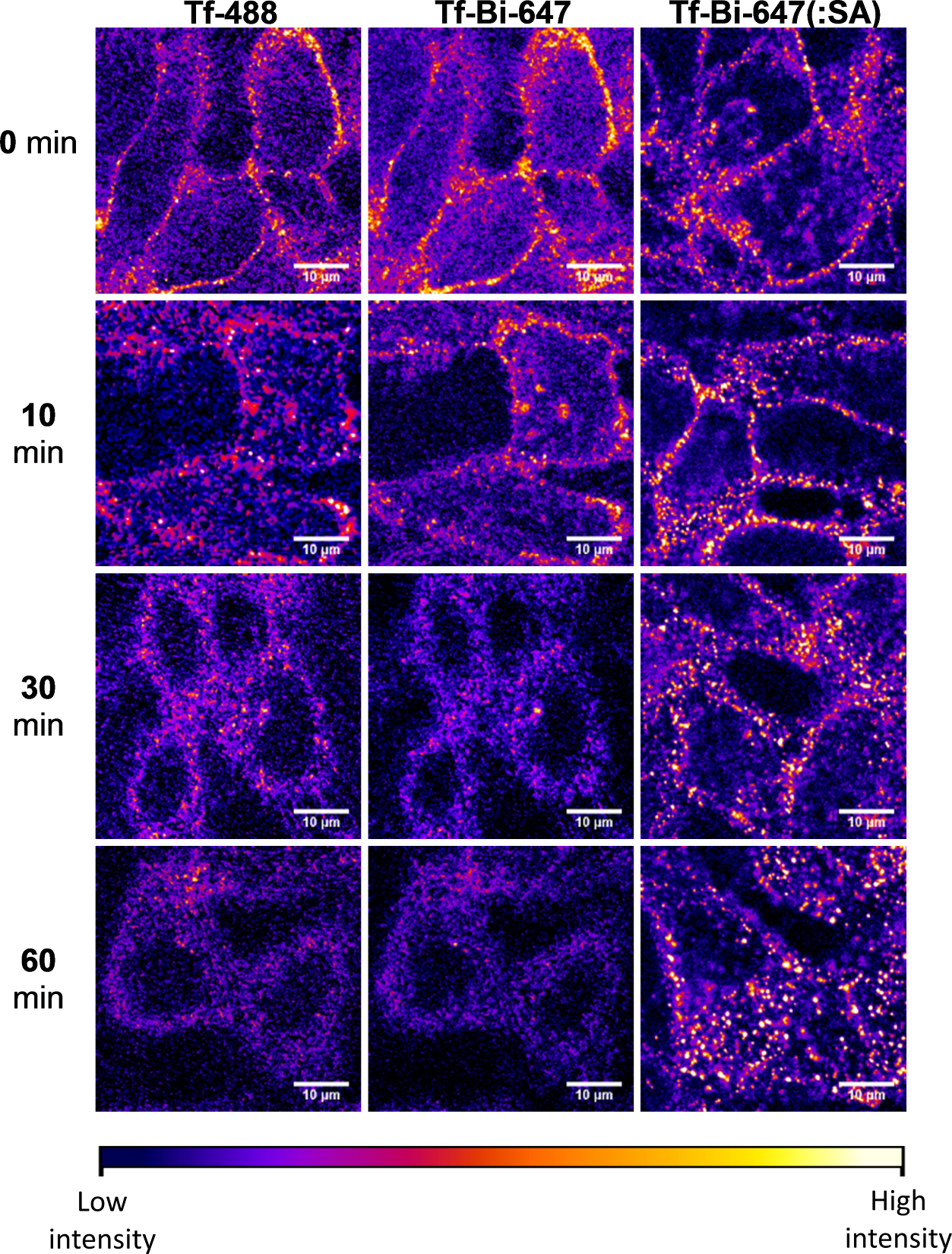
**

**Supplementary Figure S3. Recycling of Tf-488 and Tf-Bi-647 and cellular retention of Tf‑Bi-647(:SA) in HeLa cells**

To label lysosomes, cells were pulsed with 0.2 mg/ml Dex-546 for 2 hr, which was then chased at 37°C for 16 hr under tissue culture conditions. The cells were then incubated in serum-free medium for 30 min and then placed on ice for 15 min to inhibit endocytosis. Cells were then incubated for 15 min on ice with 10 µg/mL Tf-Bi-647 and 10 µg/mL Tf‑488 followed by incubation in serum-free medium (columns 1 and 2) or with 10 µg/mL Tf‑Bi‑647, followed by incubation in 1 µg/mL SA (column 3). Cells were then incubated at 37ºC in pre-warmed complete medium, and live cells were imaged by confocal fluorescence microscopy at the denoted time points. Single channel images are displayed using a “fire” lookup table, shown beneath, which illustrates the colours used to denote different pixel intensities. Representative images of Tf-Bi-647 and Tf-488 recycling are shown the presence and absence of streptavidin. These figures demonstrate that Tf-488 and Tf-Bi-647 recycle at similar rates, whilst Tf-Bi-647:SA complexes are retained in intracellular vesicles. Scale bar = 10 μm.


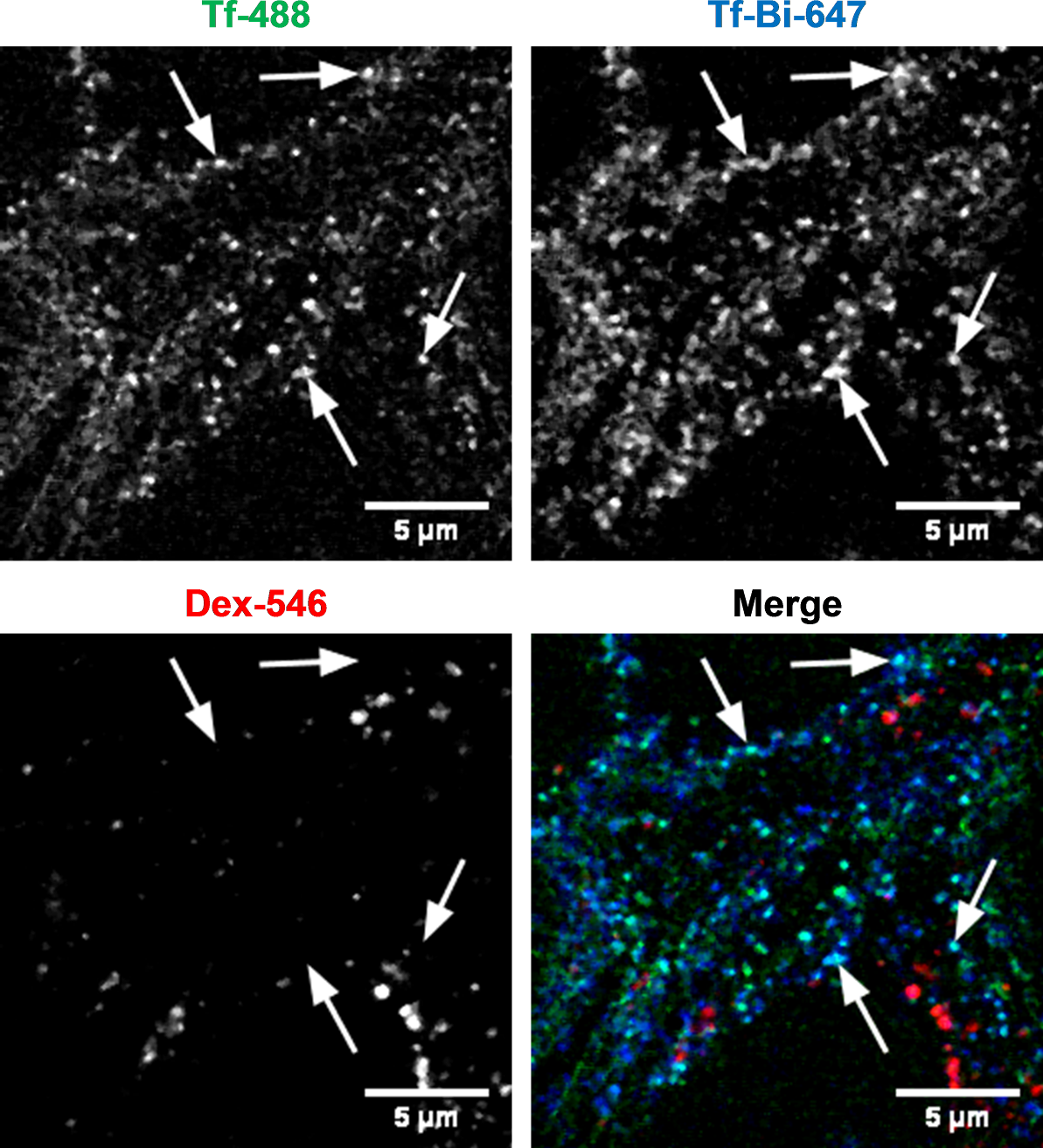


**Supplementary Figure S4. After 10 min internalisation, Tf-488 and Tf-Bi-647 colocalise together, but not with lysosomes**

To label lysosomes, cells were pulsed with 0.2 mg/ml Dex-546 for 2 hr, then chased at 37°C for 16 hr under tissue culture conditions. Cells were incubated in serum-free medium for 30 min, chilled on ice for 15 min prior to co-incubation on ice with 10 µg/mL Tf-Bi-647 and 10 µg/mL Tf‑488. Cells were then incubated at 37ºC in pre-warmed complete medium for 10 min before imaging by confocal fluorescence microscopy. Arrows denote colocalisation of Tf‑488 and Tf-Bi-647. Scale bar = 5 μm.

**a**


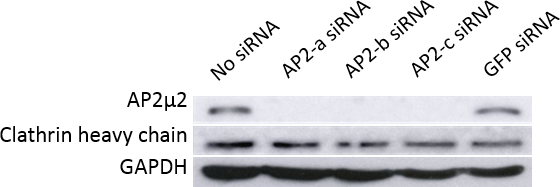


**b**

**
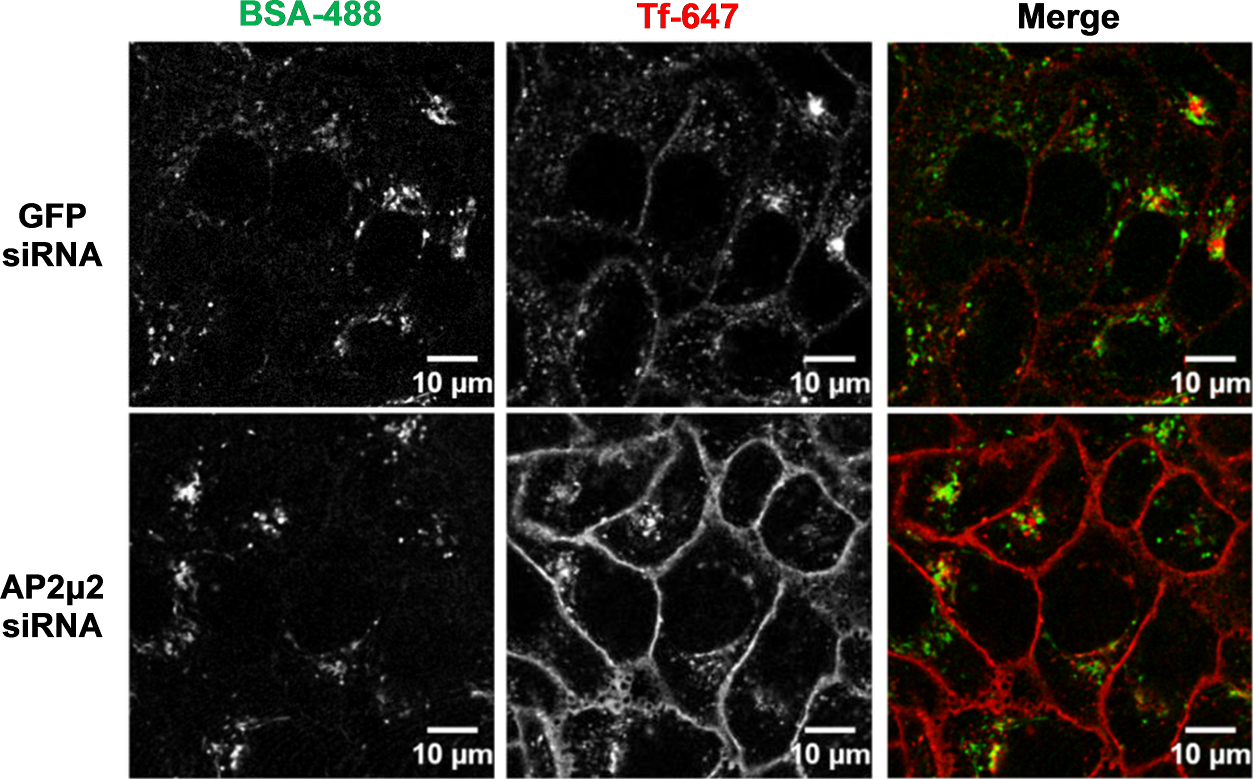
**

**Supplementary Figure S5. Depletion of AP2μ2 by siRNA and inhibition of Tf uptake in HeLa cells**

Cells were treated with control GFP siRNA^2^, or one of three siRNA sequences targeting AP2μ2: (AP2-a)^4^, (AP2-b) or (AP2-c). (a) siRNA treatment was demonstrated by Western blot to effectively deplete AP2μ2 compared with no effects on loading controls clathrin heavy chain and GAPDH. (b) After treatment with 100 pmols siRNA against either GFP or AP2μ2 (using the AP2-a, sequence) for 48 hr, HeLa cells were incubated for 30 min in serum-free medium followed by co‑incubation with 100 μg/mL BSA-488 and 10 μg/mL Tf‑647 for 15 min at 37°C. Cells were washed and the medium replaced with imaging medium and immediately imaged on a confocal microscope. These images demonstrate that depletion of AP2μ2 selectively disrupted uptake of Tf and not of BSA that has been shown to enter cells through different routes including those regulated by caveolin^5^.


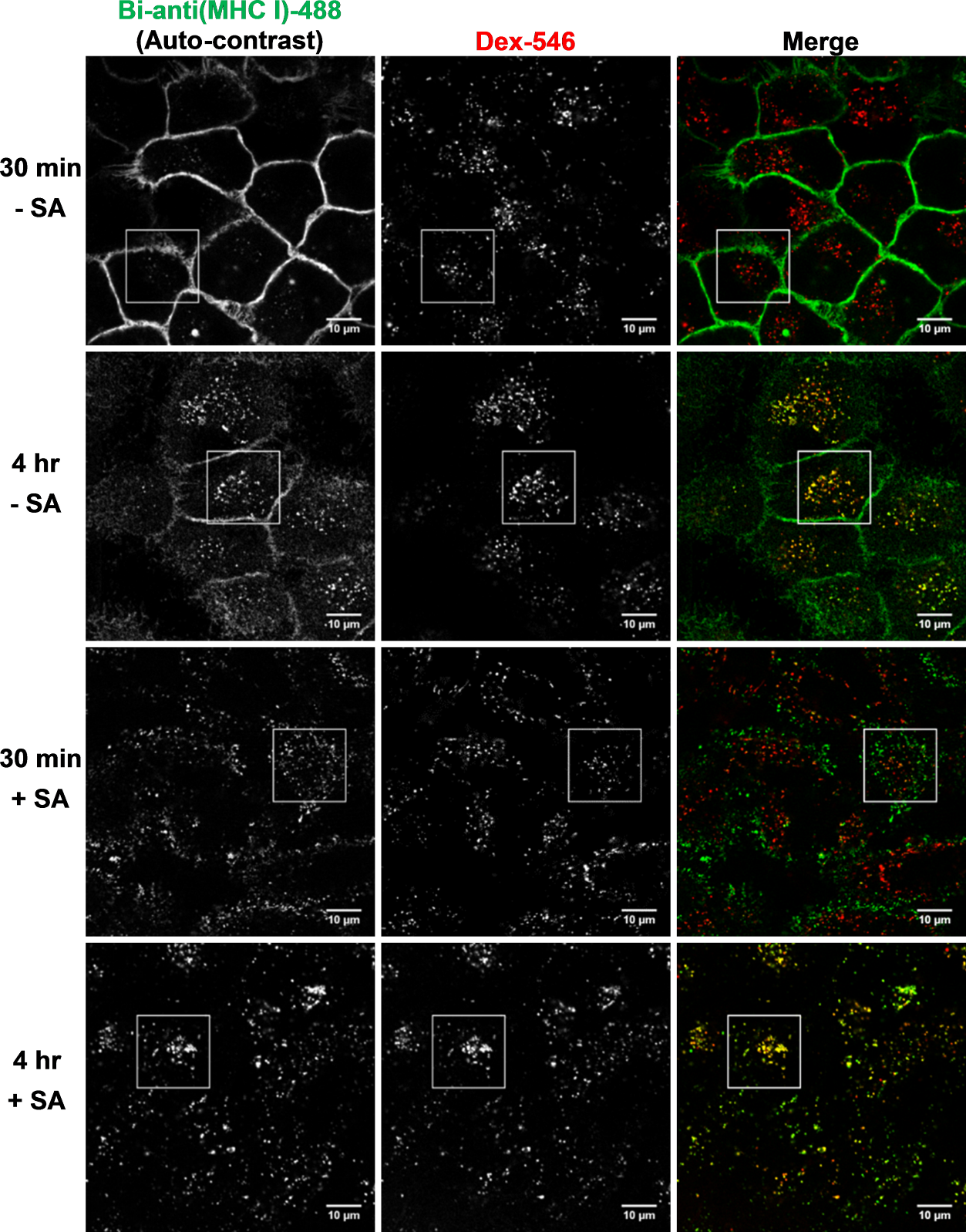


**Supplementary Figure S6. Internalisation of Bi-anti(MHC I)-488 complexes in HeLa cells is dramatically enhanced by addition of SA**

HeLa cells were pulse‑chased with Dex-546 to label lysosomes, then labelled sequentially with Bi‑anti(MHC I)-488, followed by 0 or 1 µg/mL SA. Cells were washed and then incubated at 37°C for 240 min, with live cells imaged at the denoted time points by confocal microscopy. The highlighted boxes denote the regions displayed in Figure 4a. Note that intensities in the left column have been enhanced post-acquisition and so cannot be directly compared. Representative images are shown. Scale bar = 10 μm.


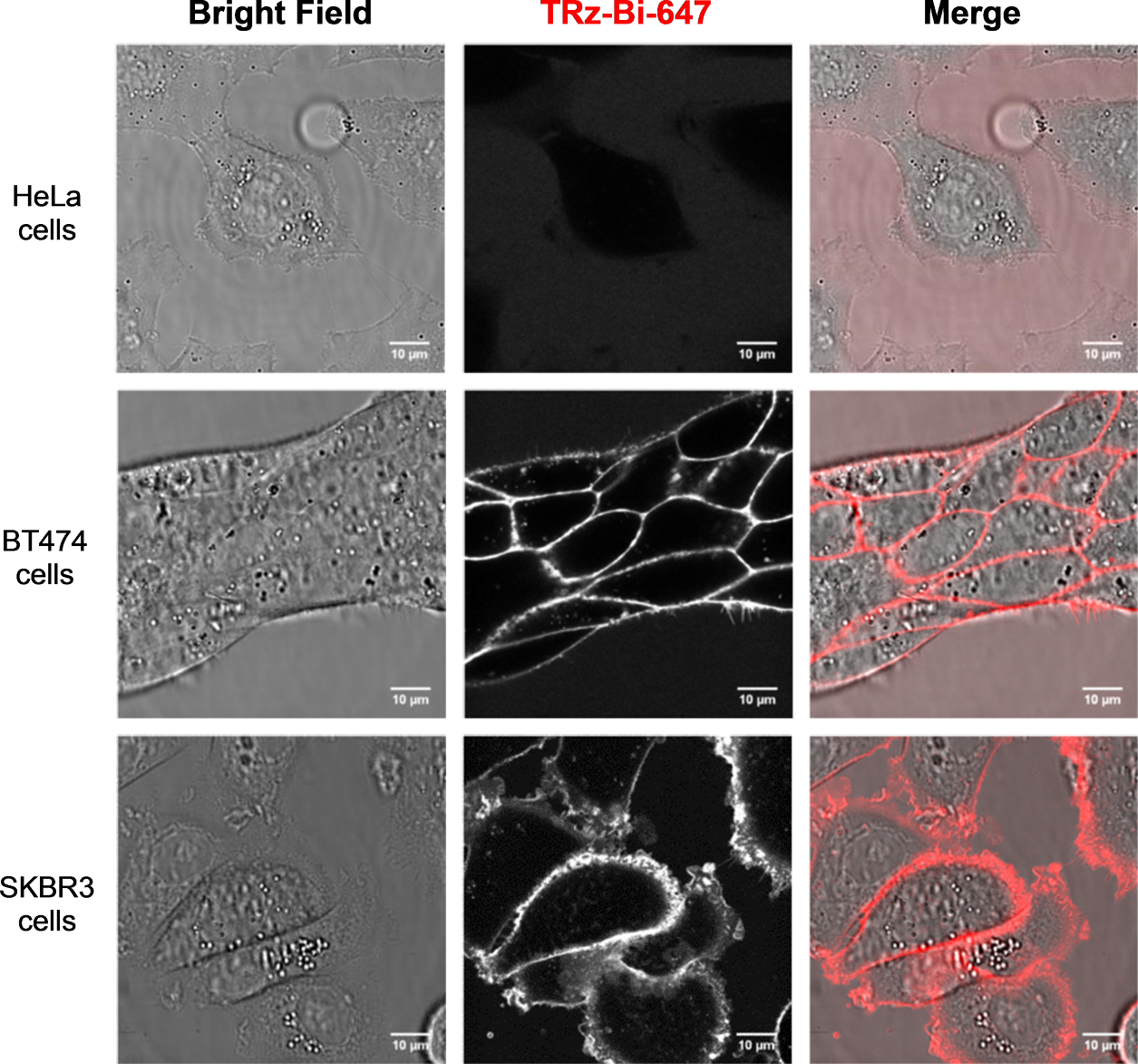


**Supplementary Figure S7. TRz-Bi-647 selectively binds to cells that express Her2**

HeLa, BT474 and SKBR3 cells were incubated with 50 nM solution of TRz-Bi-647 in complete medium at 37°C for 30 min. Live cells were then imaged in the continued presence of TRz-Bi-647 by confocal microscopy. The image contrast was set identically for each image. TRz-Bi-647 did not bind to HeLa cells, and only extracellular fluorescence is visible in this image. In contrast, TRz-Bi-647 strongly stained the membranes of Her2-expressing cells, which suggests that TRz‑Bi‑647 retains selectivity for Her2. Scale bar = 10 μm.

**
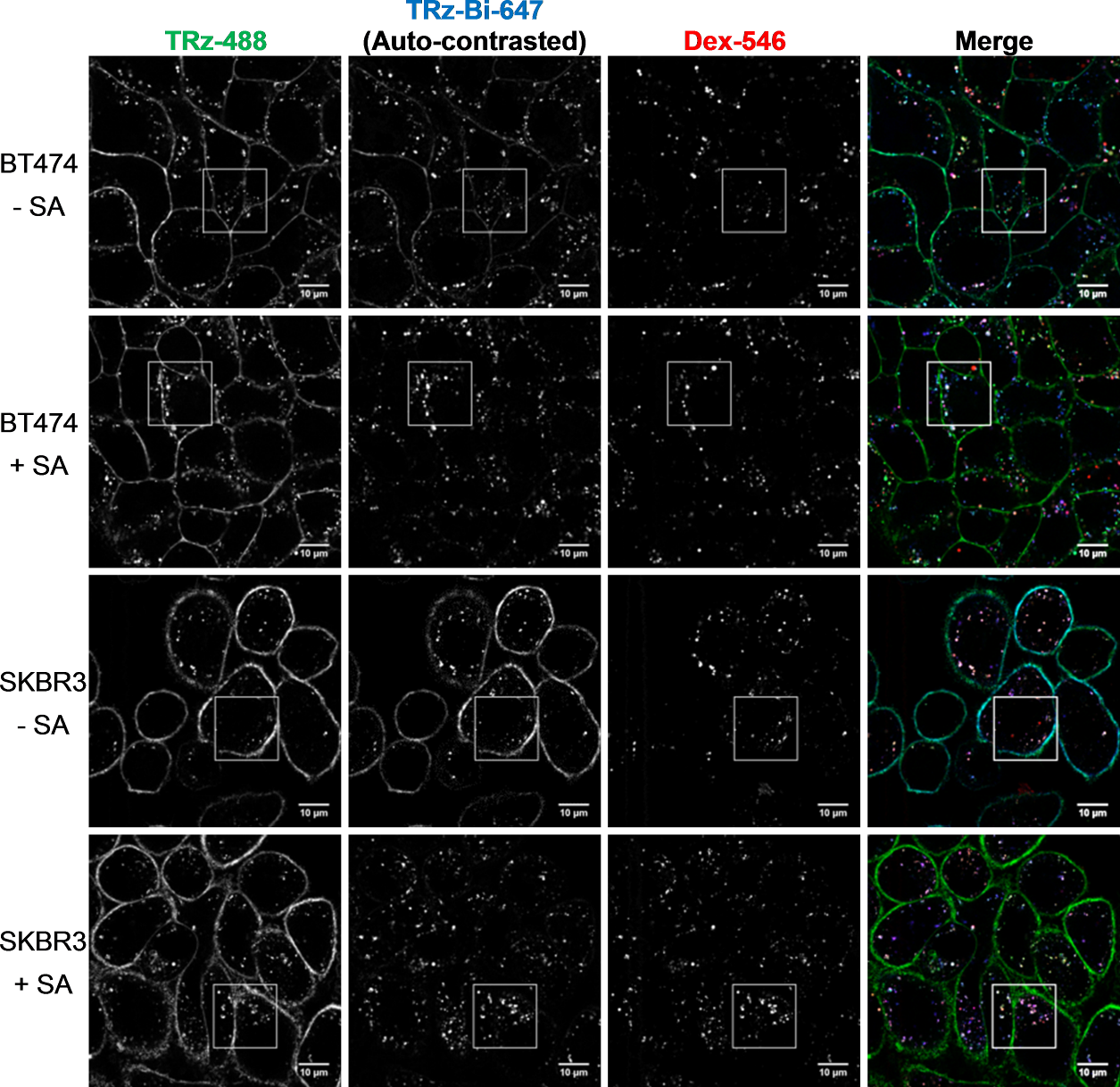
**

**Supplementary Figure S8. SA selectively increases delivery of TRz-Bi-647 to lysosomes**

Dex-546 loaded SKBR3 and BT474 cells were co-incubated at 37°C with TRz-488 and TRz‑Bi-647, then with 0 or 1 µg/mL SA prior to incubation at 37°C for 7 hr. Wash steps were included between each incubation. Single slice confocal microscopy images of the three fluorophores in live cells were then acquired. Representative fluorescence microscopy images are shown. The highlighted boxes denote the regions displayed in Figure 5a. Arrows denote colocalisation of Dex-546 with TRz-488. Contrast settings were automatically adjusted individually for each image. Scale bar = 10 μm.

**a**

**
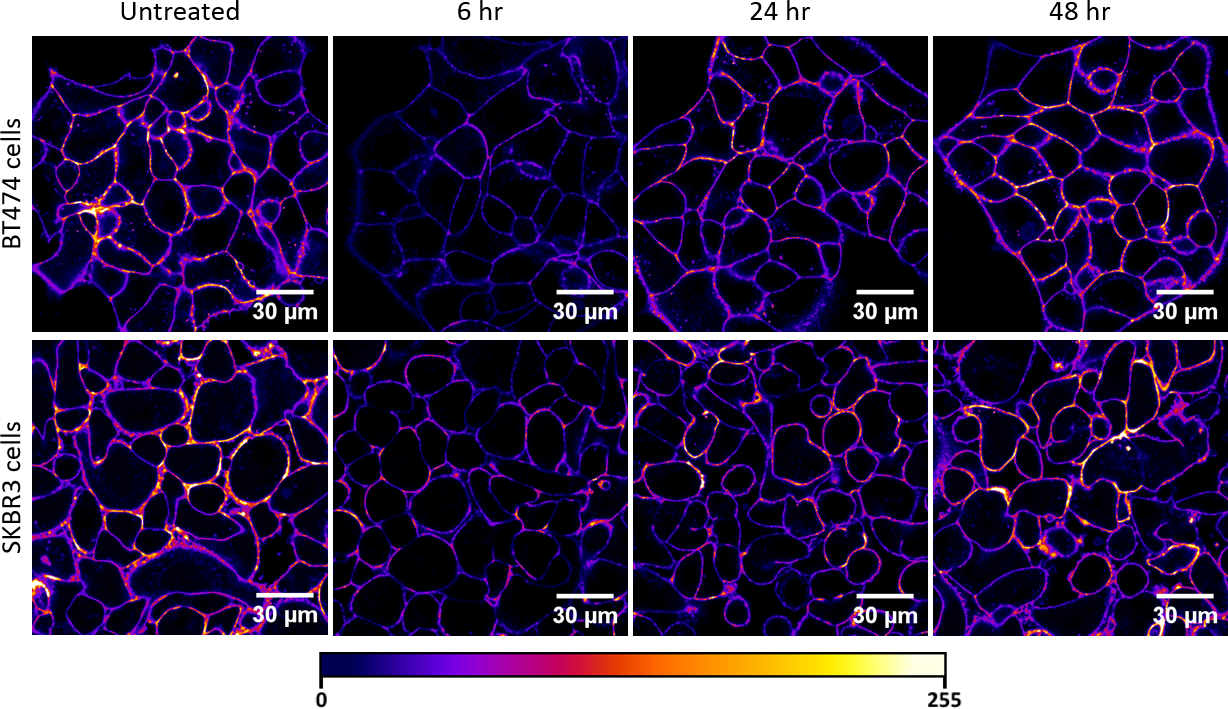
**

**b**

**
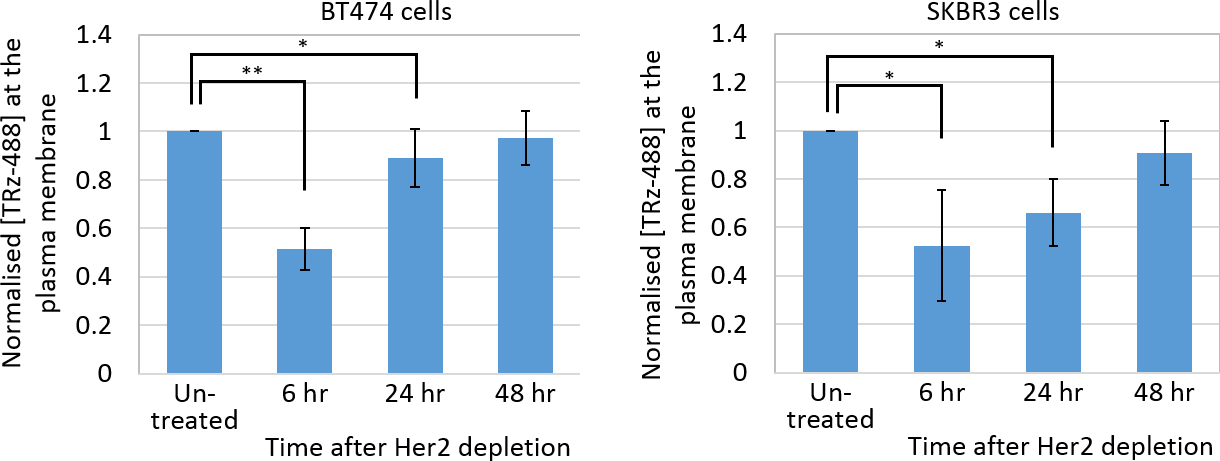
**

**Supplementary Figure S9. Rate of recovery of Her2 at the plasma membrane, following depletion with TRz-Bi-647 and SA**

SKBR3 and BT474 cells were incubated at 37°C with 1 µg/mL TRz-Bi-647 (or not treated), then with 1 µg/mL SA (or not treated), prior to incubation at 37°C for the indicated time. All cells were then incubated with 1 µg/mL TRz-488 for 1 hr prior to imaging. Single slice confocal micrographs were obtained of live cells as described in Supplementary Methods. (a) Representative fluorescence microscopy images of TRz-488 fluorescence (single channel) are shown, which are displayed using a fire lookup table. Contrast settings were automatically adjusted equally for each image post-acquisition. Scale bar = 30 μm. (b) Images from 3 independent experiments (N=3) were quantified, and Normalised Intensity values were calculated for TRz-488. p-values were calculated using a 1-tailed paired student’s t-test.
*p < 0.05, **p < 0.01. Scale bar = 30 μm.

**Supplementary Material - References**

1. Li, CH and Lee, CK (1993). Minimum cross entropy thresholding. *Pattern Recognit.* **26:** 617–625.

2. Al-Soraj, MH, Watkins, CL, Vercauteren, D, De Smedt, SC, Braeckmans, K and Jones, AT (2010)*.* siRNA versus pharmacological inhibition of endocytic pathways for studying cellular uptake of cell penetrating peptides. *J. Controlled Release* **148:** 86–87.

3. James, NG and Mason, AB (2008). Protocol to determine accurate absorption coefficients for iron containing transferrins. *Anal. Biochem.* **378:** 202–207.

4. Motley, A, Bright, NA., Seaman, MNJ and Robinson, MS (2003). Clathrin-mediated endocytosis in AP-2-depleted cells. *J. Cell Biol.* **162:** 909–918.

5. Singh, RD, Puri, V, Valiyaveettil, JT, Marks, DL, Bittman and R, Pagano, RE (2003)*.* Selective caveolin-1-dependent endocytosis of glycosphingolipids. *Mol. Biol. Cell* **14:** 3254–3265.
